# Factors associated with progression of fibrosis in chronic hepatitis B virus infection in the indeterminate phase

**DOI:** 10.12688/f1000research.157075.1

**Published:** 2025-01-02

**Authors:** Sana Rouis, soumaya mrabet, Mohamed Ferjaoui, Nedia Ben Lasfar, Jihene Sahli, Syrine Boujamline, Rym Ayari, Maha Abid, Manel Ben Selma, Mariem Ben Ticha, Foued Bellazreg, Elhem Ben Jezia, Amel Letaief, Wissem Hachfi

**Affiliations:** 1 Department of Infectious Diseases, Faculty of Medicine of Sousse, University of Sousse, Ibn El Jazzar University Hospital, Kairouan, Tunisia; 2 Department of Hepato-gastroenterology, Faculty of Medicine of Sousse, University of Sousse, Farhat Hached University Hospital, Tunisia; 3 Department of Infectious Diseases, Faculty of Medicine of Sousse, University of Sousse, Farhat Hached University Hospital, Tunisia; 4 Department of Familial and Community Medicine, Faculty of Medicine of Sousse, University of Sousse, Sousse, Tunisia; 5 Faculty of Medicine of Sousse, University of Sousse, 4000, Sousse, Tunisia

**Keywords:** hepatitis b virus, indeterminate phase, liver fibrosis, risk factors

## Abstract

**Background:**

Anti-viral therapy is not routinely recommended for chronic hepatitis B virus (HBV) infection, in patients who have persistently elevated serum HBV DNA level (>2000 IU/mL), normal alanine aminotransferase (ALAT) and without significant liver fibrosis, defining the indeterminate phase. The objective of the study is to identify the factors associated with the progression of liver fibrosis in chronic HBV infected patients in the indeterminate phase.

**Methods:**

This is cross-sectional study, conducted in Infectious Disease and Hepato-gastroenterology departments of Farhat Hached university hospital, between January 2008 and January 2022. We have included the Ag HBs (+) patients initially not treated, presenting at the time of the initial evaluation: a viral load> 2,000 IU/L for at least six months, normal ALAT (<40UI) and a fibrosis score F0 and/or F1 (in liverbiopsy or FibroScan). Univariate and logisticreg ression analysis were performed to identify the factors associated with liver fibrosis progression.

**Results:**

In total, 97 patients were included, with a median age of 32.9± 9.1 years, and a female predominance (sex ratio M/F=0.64). Progression of fibrosis was observed in 16 patients (16.5%) with a mean delay of 70.9±41.1 months. In the univariate analysis, factors associated with progression of fibrosis were the presence of comorbidities (p=0.001), the high initial viral load (p=0.004), the appearance of cytolysis (p=0.001) and the increase in viral load (p=0.002), during follow-up. The AUROC of the initial viral load was 0.664 (95%CI: 0.500-0.820). An intial viral load at 8090 UI/l was associated with the progression of fibrosis with a sensibility of 70.3% and specificity of 63%.

**Conclusion:**

Factors associated with progression of fibrosis in the indeterminate phase of chronic HBV infection were the presence of comorbidities, and changes of ALAT during follow-up. This leads us to consider extending the therapeutic indications to this group of patients.

## Introduction

Infection with hepatitis B virus (HBV) is a public health problem with significant morbidity and mortality associated with cirrhosis and its complications. ^1^ According to the 2017 World Health Organization (WHO) global report, more than two billion people have been exposed to HBV. ^2^ In North Africa, HBV infection has been described as a major etiological agent for the development of hepatocellular carcinoma (HCC). ^3^ In Tunisia, the national prevalence of HBs Ag 1.7%. ^4^

Chronic HBV infection is a dynamic process that reflects the interaction between HBV replication and host immune response. According to national and international recommendations, the indication for antiviral treatment in chronic hepatitis B (CHB) is based primarily on the combination of the following three criteria: viral load of HBV, ALAT levels, and severity of liver histological lesions. Thus, antiviral treatment is indicated only in patients with a viral load> 2,000 IU/L, whether or not associated with cytolysis and with significant hepatic fibrosis (≥F2) on liver biopsy or elastography. However, in the presence of a viral load> 2000 IU/L, there is a risk of disease progression to cirrhosis and HCC. ^5^ Untreated patients should be monitored by regular transaminase and B viral load determinations, as well as by noninvasive fibrosis assessment and liver ultrasound. However, the modalities of monitoring its rhythm are not well established, and there are few data concerning the natural history of these patients. ^1^

Patients with a viral load >2,000 IU, but who do not meet the criteria defining therapeutic indications, have recently been classified in the so-called “indeterminate phase”. ^5– 7^ They present an increased risk of progression of hepatic fibrosis, which has been objectified on the basis of noninvasive markers of fibrosis, such as the FIB-4 Score. This progression was on the order of 11% per year in the absence of certain factors, such as advanced age, metabolic syndrome, and chronic ethylism. ^8^

The virosuppression achieved by anti-viral therapy in chronic hepatitis B reduces the risk of progression to fibrosis and HCC. Anti-viral treatment with analogues has been shown to prevent around 60% of new cases of HCC over a 10-year period. ^9^

However, other studies have shown that the risk of fibrosis progression, cirrhosis, and its complications is minimal and comparable between treated and untreated patients. ^10– 14^

In this study, we aimed to identify the factors associated with fibrosis progression in patients with chronic HBV infection in the indeterminate phase.

## Methods

### Study design

This cross-sectional study was conducted in the Infectious Diseases and Hepato-Gastroenterology departments of Farhat Hached University Hospital between January 2008 and January 2022.

### Study setting and participants

We included all patients with chronic HBV infection in the “indeterminate phase” with Ag HBs (+) not initially treated, presenting at the time of initial evaluation a viral load> 2,000 IU/for at least six months, normal ALAT (< 40 IU), and fibrosis score F0 and/or F1 (on liver biopsy or FibroScan).

We did not include patients with cirrhosis, co-infection with hepatitis D virus (HDV), hepatitis C virus (HCV), or human immunodeficiency virus (HIV), a personal or family history of HCC, patients treated at the time of diagnosis, immunotolerant patients, and those receiving pre-emptive treatment during follow-up.

### Data analysis

Data were collected from the medical records of patients using a standardized data form. In fact, data collection began at the start of the cohort in January 2008. Data processing began in May 2023, when the team started the study. The baseline data comprised sociodemographic details, including age, sex, profession, and address. We also considered comorbidities such as arterial hypertension, dyslipidemia, diabetes mellitus, chronic alcoholism (two to three standard glasses per day), body mass index (classified into 4 categories: <25 kg/m ^2^/25-30/30-35/ >35 kg/m ^2^), hepatic steatosis (on abdominal ultrasound and/or liver biopsy), HBe Ag (positive or negative), initial alanine aminotransferase (ALAT) rate (normal is defined as < 40 IU/L), and initial viral load (over the course of the disease, classified into 2 categories:moderate (2000–20,000 IU/L) and high (>20,000 IU/L).

During the follow-up, the viral load was monitored (increasing/decreasing/fluctuating), ALAT rate (/6 months) (normal/high/fluctuating), METAVIR score at liver biopsy control, and liver stiffness at elastography (FibroScan) control. The following events were noted: progression of fibrosis, cirrhosis, and HCC.

The indications for antiviral treatment during follow-up included the progression of fibrosis (defined by an increase of at least 1 point in METAVIR score on liver biopsy, ^1^ or an increase in liver stiffness of at least 1 kPa on FibroScan), progression to cirrhosis (defined by a METAVIR F4 score on FibroScan, or an elasticity greater than 15 kPa on FibroScan and/or the presence of indirect clinical, biological, and morphological signs), the occurrence of HCC (for which diagnosis is based on hepatic angioscan or MRI data, or on histological data from liver nodule biopsy), HBs or HBe seroclearance (to look for loss of HBs or HBe Ag), HBs, or HBe seroconversion (appearance of Anti HBs or Anti HBe antibodies).

### Statistical analysis

Statistical analysis was performed using IBM SPSS 20.0 software ( https://www.ibm.com/fr-fr/products/spss-statistics ).

Quantitative variables are expressed as the mean ± SD. Qualitative variables are expressed as percentages. The comparison of two means on independent series was carried out using Student’s t-test. The comparison of percentages was performed using the Pearson χ ^2^ test and, in the event of non-validity, using the exact two-sided Fisher test. Associations between the different variables and liver fibrosis progression were estimated in a univariate analysis and a logistic regression analysis with the expression of an odds ratio (OR) with a 95% confidence interval. The relevant factors were analyzed, and their diagnostic value was evaluated using the receiver operating characteristic (ROC) curve and the area under the ROC curve (AUROC). For all statistical tests, statistical significance was set at p<0.05.

## Results

### Enrolled patients

**Figure 1.**
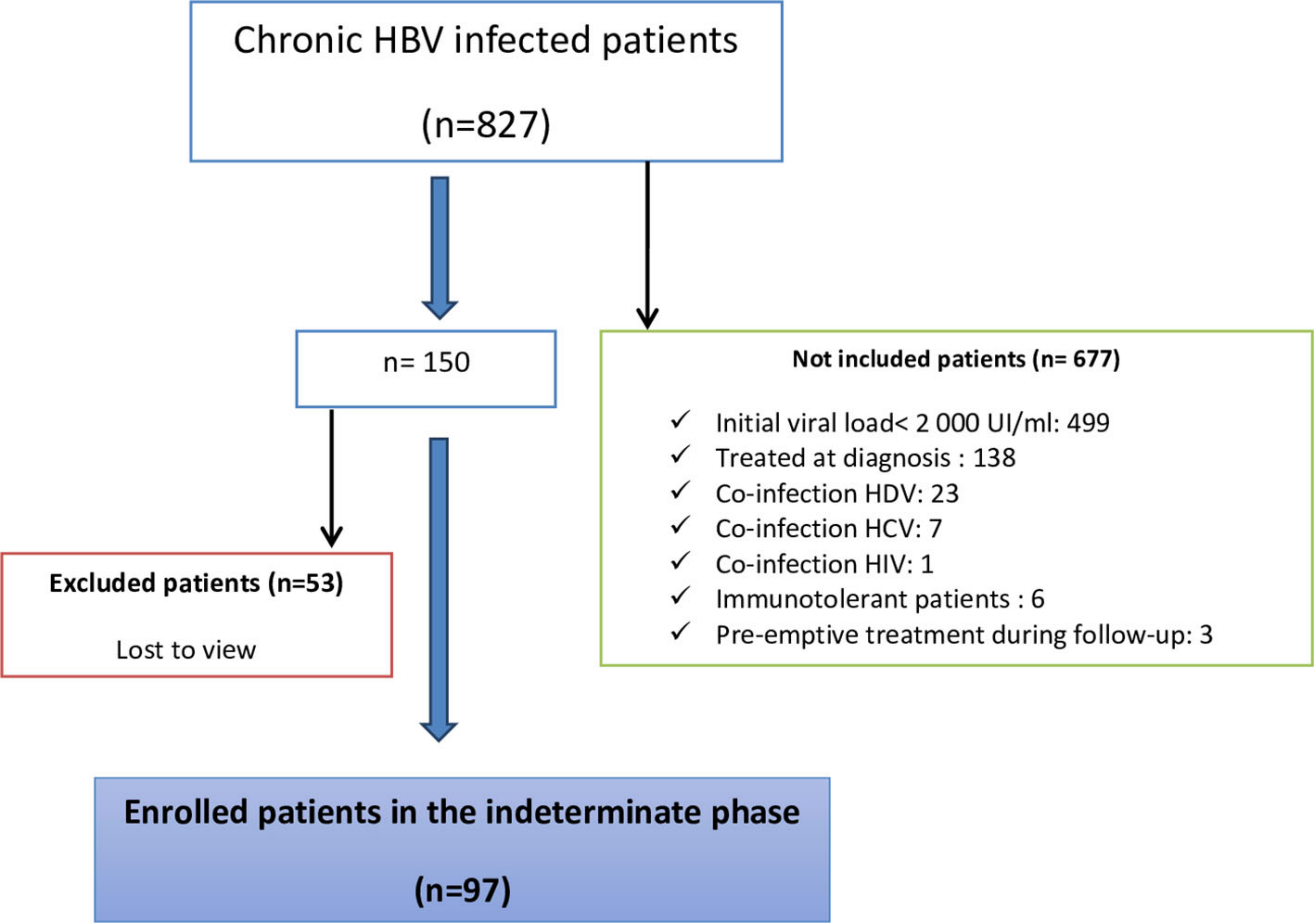
The flow chart of the study population.

A total of 827 patients were followed up for chronic HBV infection. There were 53 patients who were excluded because they were lost to follow-up. The flow diagram of the study population is shown in Figure 1. Moreover, 677 patients were not included in the study; among them, 499 had an initial viral load of < 2000 UI/ml. Finally, 97 patients who fulfilled the inclusion criteria were included ( Figure 1).

### Baseline characteristics

The baseline characteristics of the included patients are shown in Table 1. The mean age of the participants was 32.9 ± 9.1 years. The mean initial viral load was 8 182 122.4 UI/ml. Among these patients, 38 (39.2%) were men (sex ratio M/F=0.64) and 95 (97.9%) were HBeAg-negative.

**Table 1.** Baseline Characteristics of enrolled patients withchronic HBV infection in the indeterminate phase (n = 97).

Variables		n (%)
**Age, mean± SD (years**)	32.9 ± 9.1	
< 20 years old		6 (6.2)
20-40 years old		72 (74.2)
40-60 years old		18 (18.6)
>60 years old		0 (0.0)
**Gender, n (%)**		
Male		38 (39.2)
Female		59 (60.8)
**Underlying comorbidities, n (%)**		13 (13.4)
Dyslipidemia		7 (7.2)
Diabetesmellitus		4 (4.1)
High blood pressure		2 (2.1)
**BMI, mean± SD (kg/m** ^**2**^ **)**	26.1± 4.6	
<25 kg/m ^2^		30 (30.9)
25-30 kg/m		32 (33.0)
30-35 kg/m ^2^		9 (9.3)
>35 kg/m ^2^		2 (2.1)
**HbeAg, n (%)** Positive		2 (2.1)
Negative		95 (97.9)
**Initial viral load**, **mean±SD (UI/l)**	8 182 122.4 ± 31488242.1	
Moderate, n (%)		81 (83.5)
High, n (%)		16 (16.5)
**Initial fibrosis, n (%)**		62 (60.2)
Liver biopsy		40 (41.2)
FibroScan		57 (58.8)

Underlying comorbidities were observed in 13.4% of patients (n=13). These comorbidities included dyslipidemia (n=7), diabetes (n=4), and high blood pressure (n=2). The mean Body Mass Index (BMI) was 26,1± 4.6 kg/m ^2^. The initial assessment of fibrosis was performed in all patients (liver biopsy (41.2%) and elastography (58.8%)) ( Table 1). The mean initial liver stiffness was 4,9± 1.2 kPa.

### Outcomes

The median follow-up duration was 105.2 ± 48.4 months. Progression of fibrosis was observed in 16 patients (16.5 %), with a mean delay of 70.9±41.1 months. Antiviral treatment was initiated. Complications that occurred in the enrolled patients included cirrhosis (n=3), HCC (n=1), and death (n=1). Serologically, 11 patients (10.7%) had a loss of HBsAg and five had a loss of HBe Ag (4.9%), with a mean delay of 87± 49 and 51± 24.7 months ( Table 2).

**Table 2.** Outcomes of enrolled patients with chronic HBV infection in the indeterminate phase (n = 97).

Variables		n (%)
Changes in ALAT, n (%)	Normal	86 (88.7)
	Fluctuatingcytolysis	10 (10.3)
	Persistent cytolysis	1 (1.0)
Viral load during follow-up, n (%)	low	33 (34.0)
	moderate	10 (10.3)
	High	7 (7.2)
	flucuating	47 (48.5)
Direction of evolution of viral load, n (%)	Stable	8 (8.2)
	Increasing	8 (8.2)
	Decreasing	26 (26.8)
	Fluctuating	55 (56.7)
Liver Fibrosis, n (%)	Stable	81 (83.5)
	Progression	16 (16.5)
	Regression	0 (0.0)
Use of antiviral treatment during follow-up, n (%)		16 (16.5)
Changes in serological profile, n (%)	Loss of HBs Ag	10 (10.3)
	Seroconversion HBs	8 (8.2)
	Loss of HBe Ag	2 (2.1)
	Seroconversion HBe	2 (2.1)
Complications, n (%)	Cirrhosis	3 (3.1)
	HCC	1 (1.0)
	Death	1 (1.0)

### Risk factors associated with liver fibrosis

**Figure 2.**
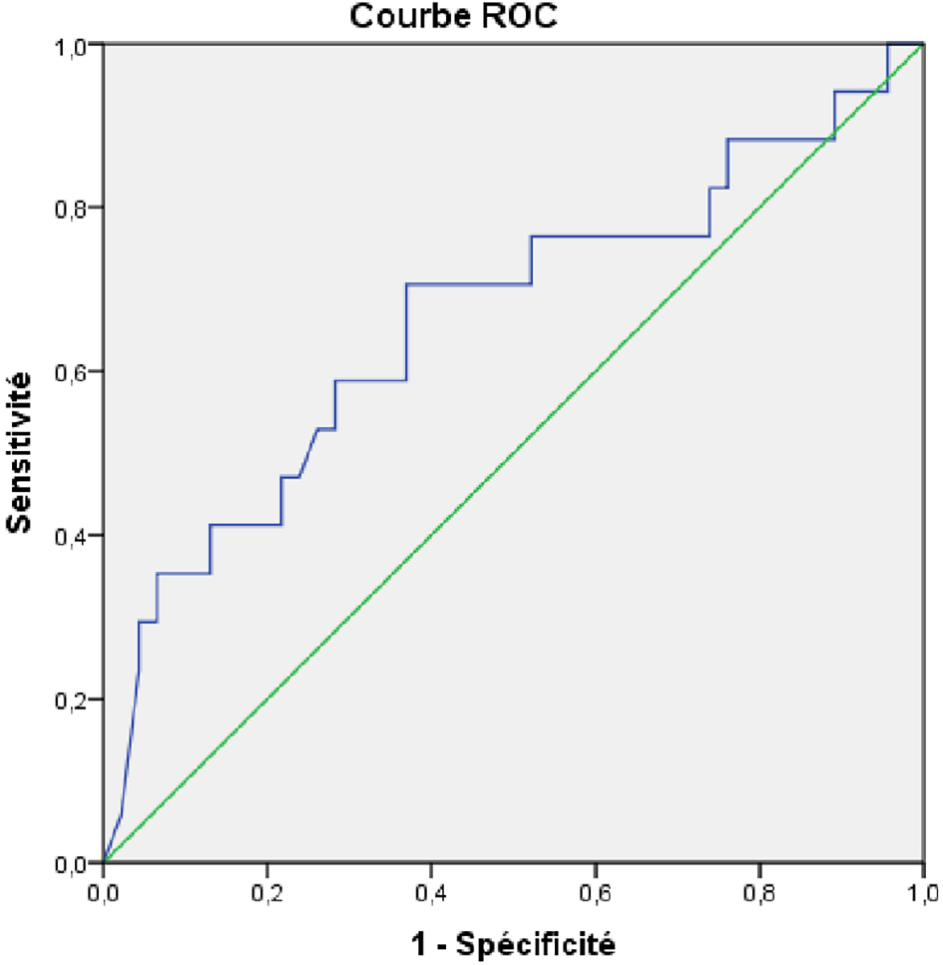
AUROC of the initial viral load as a factor associated with liver fibrosis progression in patients with chronic HBV infection in the indeterminate phase.

In the univariate analysis, factors associated with the progression of fibrosis were the presence of comorbidities (p=0.001), high initial viral load (p=0.004), appearance of cytolysis (p=0.001), and increased viral load (p=0.002) during follow-up ( Table 3). The AUROC of the initial viral load was 0.664 (95%CI: 0.500-0.820). An initial viral load of 8090 UI/l was associated with the progression of fibrosis with a sensitivity of 70.3% and specificity of 63% ( Figure 2).

**Table 3.** Factors associated with progression of fibrosis in patients with chronic HBV infection in the indeterminate phase in the univariate analysis (n=97).

Factors	Progress of liver fibrosis	No progression of liver fibrosis	p
	N=16	N=81	
**Age, mean (years)**	33.1	31.7	0.708
< 20 years old	1 (16.7)	5 (83.3)	
20-40 years old	11 (15.3)	61 (84.7)	0.799
40-60 years old	4 (22.2)	14 (77.8)	
>60 years old	0 (0.0)	0 (0.0)	
**Sex, n (%)**			
Male	6 (15.8)	32 (84.2)	0.881
Female	10 (16.9)	49 (83.1)	
**Smoking, n (%)** yes	1 (14.3)	6 (85.7)	0.929
No	5 (15.6)	27 (84.4)	
**Alcohol, n (%)** Yes	2 (25.0)	6 (75.0)	0.486
No	13 (15.5)	71 (84.5)	
**Comorbidities, n (%)** Yes	8 (61.5)	5 (38.5)	0.001
No	8 (9.5)	76 (90.5)	
**BMI, mean (kg/m ^2^)**	24,3	26,1	0.411
**Steatosis, n(%)** Yes	4 (16.7)	20 (83.3)	0.979
No	12 (16.4)	61 (83.6)	
**Initial rate of ALAT, n** (%)			
≤40 UI/l	15 (16.1)	78 (83.9)	0.640
> 40 UI/l	1(25.0)	3(75.0)	
**HBe Ag, n (%)**			
Positive	1 (50.0)	1 (50.0)	0.197
Negative	15 (15.8)	80 (84.2)	
**Initial viral load, n (%)**			
Moderate	9 (11.1)	72 (88.9)	**0.004**
High	7(43.8)	9 (56.2)	
**Changes in ALAT, n (%)**			
Normal	9 (10.5)	77 (89.5)	0.001
Persistant cytolysis	0 (0.0)	1 (100.0)	
Fluctuatingcytolysis	7 (70.0)	3 (30.0)	
**Changes of viral load, n (%)**			
Low	0 (0.0)	33 (100.0)	
Moderate	5 (50.0)	5 (50.0)	**0.001**
High	4 (57.1)	3 (42.9)	
Fluctuating	7 (14.9)	40 (85.1)	
**Direction of evolution of viral load, n (%)**			
Stable	0 (0.0)	8 (100.0)	
Increasing	5 (62.5)	3 (37.5)	**0.002**
Decreasing	2 (7.7)	24 (92.3)	
Fluctuating	9 (16.4)	46 (83.6)	

In the multivariate analysis, the factors associated with fibrosis progression were the presence of comorbidities (Odds Ratio (95% CI)=53.345 (8.612-330.437), p<0.001) and the changes in ALAT rates (odds ratio (95% CI)=8.539 (3.168-23.018), p<0.001), but not with the initial viral load or the changes in viral load ( Table 4).

**Table 4.** Factors associated with progression of fibrosis in patients with chronic HBV infection in the indeterminate phase in the logistic regression analysis (n=97).

Factors	Odds Ratio	(95% CI)	p
**Comorbidities, n (%)** Yes	53.345	[8.612-330.437]	**<0.001**
No		**Ref**	
**Changes in ALAT, n (%)** Normal			
Persistant cytolysis	8.539	**Ref**	
Fluctuatingcytolysis		[3.168-23018]	**<0.001**

## Discussion

The main findings of our study were as follows: First, progression of fibrosis was observed in 16 patients (16.5%), with a mean delay of 70.9±41.1 months. Second, the factors associated with the progression of fibrosis were the presence of comorbidities, high initial viral load, cytolysis, and increased viral load during follow-up. An initial viral load of 8090 UI/l was associated with the progression of fibrosis, with a sensitivity of 70.3% and specificity of 63%.

### Frequency of the indeterminate phase among chronic HBV-infected patients

Our study included chronic VHB infected patients in the indeterminate phase, which represented 11.7 % of all the patients belonging to the CHB cohort of Farhat Hached University Hospital (n=827). Although the treatment indications for chronic VHB infection are clear, more attention should be paid to those not meeting treatment indications, defining patients in the “indeterminate phase.” In our study, these patients represented 11.7% of all patients. This finding was less than that reported in a retrospective multicenter cohort study conducted in the USA and Taiwan China, ^5^ in 3366 CHB patients were followed up for at least 1 year.

The findings showed that patients in the indeterminate phase accounted for, on average, 31.8% of the Chinese and Taiwanese cohorts and 38.7% of the American cohort. Additionally, there were 4759 CHB patients in Nanjing, China, of which 27.8% were in the indeterminate phase, according to Yao et al. ^7^ The percentage of patients in the indeterminate phase found in our study may be explained byour younger population with a predominance of inactive carriers among all the VHB-infected patients followed in our center.

### Factors associated with liver fibrosis progression

Jiang et al.reported that 24.3% of patients in the indeterminate phase are at risk of disease progression. ^15^ In our study, among the 97 patients, 16 (16.5%) developed fibrosis, leading to the initiation of antiviral treatment. In a previous study that included 234 patients with CHB who did not meet the treatment criteria at presentation and during a median follow-up period of 51 months, 19.2% of patients transitioned to a more active disease phase and 18.8% started antiviral therapy. ^16^ Huang et al. reported that among 1303 patients in the indeterminate phase, 283 (21.7%) transitioned to immune-active disease by up to 10 years of follow-up evaluation. ^5^

In our study, in the univariate analysis, factors associated with the progression of fibrosis were the presence of comorbidities, a high initial viral load, the development of cytolysis, and the increase in viral load during follow-up. In logistic regression analysis, the factors associated with liver fibrosis progression were the presence of comorbidities and changes in ALAT rates.

A review of the literature revealed only one study that examined the factors associated with fibrosis in patients in the indeterminate phase of the disease. In this retrospective cohort study involving 634 patients with CHB infection in the indeterminate phase, ^15^ the authors found that the statistically significant variables that could affect liver fibrosis were a low/moderate HBV DNA level at the initial assessment and an increased gamma-glutamyl transpeptidase (GGT) level. In contrast, increased aspartate transaminase to platelet ratio index (APRI) and (liver inflammation and fibrosis 5) LIF-5 values ^17, 18^ were independent risk factors for liver fibrosis in the indeterminate phase. ^15^ This study showed that regardless of ALAT values, patients with an initial low/moderate viral load had more severe liver disease, in contrast to our findings. In fact, a high viral load leads to dysfunction of HBsAg-specific cytotoxic T lymphocytes, resulting in immune tolerance. However, during prolonged reproduction, HBV interacts with the host immune system and induces cumulative immune damage and, consequently, liver damage. ^15^ No other studies have analyzed comorbidities as an associated factor with the progression of fibrosis or other evolutionary parameters, such as the development of cytolysis in patients in the indeterminate phase. In fact, patients with comorbidities are not often included in studies evaluating hepatic fibrosis in HBsAg-positive patients because the progression of fibrosis may be linked to metabolic dysfunction-associated liver disease.

On the other hand, we found that an initial viral load of 8090 UI/l was associated with the progression of fibrosis, with an AUROC of 0.664 (95%CI: 0.500-0.820), a sensitivity of 70.3%, and a specificity of 63%. In the same study by Jiang et al., ^15^ low/moderate viral load was an independent factor for liver fibrosis, with an AUROC of 0.799 (95%CI: 0.760–0.838) without defining a specific cut-off. Chen et al. showed that serum HBV DNA levels in patients in the indeterminate phase were significantly higher in those with advanced inflammation and fibrosis. ^19, 20^ Elevated serum HBV DNA levels are a risk factor for significant liver inflammation in patients with CHB, which is consistent with the findings of other studies. ^21, 22^ Further studies are needed to determine the cut-off viral load associated witha high risk of fibrosis progression in patients in the indeterminate phase.

### Occurence of HCC and cirrhosis

In the present study, only one patient developed HCC (1 %) and 26 (2%) in Hunag’s study. ^5^ In fact, the correlation between viral load and the progression of end-stage liver disease (such as HCC) remains controversial. In a recent meta-analysis, the pooled annual HCC incidence was 2.54 cases per 1.000 person years (95% CI, 1.14–4) for patients in the indeterminate phase. ^23^

According to Huang et al., In addition to age 45 years and older (aHR, 20.8; 95% CI, 2.8–156.7; p=0.003), the indetermined period was independently linked to a higher risk of HCC development (aHR, 14.1; 95% CI, 1.3–153.3; p=0.03) than the inactive phase. ^5^ Contrary to the findings reported by Lee et al., in which the authors assessed the untreated persistently elevated serum HBV patient group (patients in the indeterminate phase) and analyzed the cumulative HCC risk at 3, 5, 7, and 9 years (n = 67), which were 0%, 0%, 2.9%, and 2.9%, respectively. ^11^

In contrast, we found that three patients developed cirrhosis (3.1%). In a study by Yapali et al., which included 234 patients who did not meet the criteria for antiviral treatment at presentation, none of the patients experienced cirrhosis during the follow-up. ^16^ These results are in contrast to those of Huang et al., who found a higher 10-year cumulative incidence of cirrhosis among indeterminate patients who remained indeterminate versus inactive patients who remained inactive, 8.8% (95% CI, 6.5–11.8) vs 3.5% (95% CI, 2.5–5.0; p<.0001). ^5^

### Antiviral treatment in patients with indeterminate phase

Antiviral treatment indications are generally provided to individuals at a high risk of disease progression, namely those with elevated ALAT levels, active viral replication, and advanced fibrosis or cirrhosis. ^1^

As reported above, Huang et al. found that, without treatment, 21.7% of patients in the indeterminate phase had fibrosis progression and became immune active. These patients had a higher 10-year cumulative incidence of cirrhosis than those in the inactive phase and a 14 times higher risk of HCC development. ^5^ Similarly, another observational study including 5414 patients, demonstrated that, compared to patients receiving oral antiviral therapy in the active phase, untreated HBeAg-negative CHB patients in the indeterminate phase had a considerably greater risk of HCC and mortality. ^24^ As long as HBV DNA is found, several experts have suggested that therapy should start as soon as feasible to lower the chance of the disease progressing. ^25^ Therefore, Zhou et al. recommended that antiviral therapy should be initiated in HBeAg-negative patients with normal ALAT and HBV DNA ≥ 2 000 IU/mL. ^25^ Based on our findings, we propose to treat patients with chronic HBV infection in the indeterminate phase from a viral load value of 8090UI/l, with underlying comorbidities and developing cytolysis during flollow-up. However, the clinical benefits of antiviral therapy in this population need to be confirmed in future studies.

### Limitations

To our knowledge, this is the first large-scale cross-sectional study of Tunisian patients with chronic HBV infection in the indeterminate phase. However, our study has some limitations. First, many patients were lost to follow-up and were excluded from the final analysis. Second, this study did not determine the HBV genotypes. The dominant genotype of HBV in Tunisia is genotype D, ^26^ and it has been demonstrated that genotype C infections are more prone to progress to HCC earlier, which goes some way explains the low frequency of HCC in our patients. ^22^

## Conclusions

In summary, fibrosis progression occurred in 16.5% of the patients with chronic HBV infection in the indeterminate phase. The main risk factors associated with liver fibrosis were the presence of comorbidities, high initial viral load with a cut-off of 8090 UI/l, the appearance of cytolysis, and an increase in viral load during follow-up. Further studies are required to determine whether early antiviral treatment can reduce the incidence of cirrhosis and HCC in these patients.

## Ethics and consent

This study was conducted in accordance with the standards of ethics of the research. Anonymity and data confidentiality were guaranteed for all patients and written informed consent for participation in the study was obtained. At the time of data collection, the study was designed as a retrospective review of existing patient records, which did not initially anticipate publication or require additional interventions beyond routine clinical care. Consequently, an ethical approval was not sought prospectively. However, prior to manuscript submission for several months, we obtained ethical clearance retrospectively from the Ethical Committee of the Faculty of Medicine of Sousse, Tunisia on January 10, 2024, which reviewed and approved the use of the data for research purposes [Ethical Committee Number AVIS Number 220 (Ref: CEFMS 220/2024)].

## Data Availability

The project contains the following underlying data:
• [Figshare]: sana rouis (2024). [Factors associated with progression of fibrosis in chronic hepatitis B virus infection in the indeterminate phase]. figshare. https://doi.org/10.6084/m9.figshare.28007054 Data_VHB. Dataset. ^27^ • [Figshare]: sana rouis (2024).[Factors associated with progression of fibrosis in chronic hepatitis B virus infection in the indeterminate phase] (data collection sheet). https://doi.org/10.6084/m9.figshare.27172914
• [Figshare]: sana rouis (2024). Factors associated with progression of fibrosis in chronic hepatitis B virus infection in the indeterminate phase (patient consent form). https://doi.org/10.6084/m9.figshare.27172956
•
[Figshare]: sana rouis (2024) [Factors associated with progression of fibrosis in chronic hepatitis B virus infection in the indeterminate phase] (patient information letter). https://doi.org/10.6084/m9.figshare.27902625.v1 Data are available under the terms of the Creative Commons Attribution 4.0 International license (CC-BY 4.0). Our article answers the STROBE checklist for cross-sectional studies.

## Software availability statement

The data analysis was conducted using Jamovi version 2.3, which is open-access software available at https://www.jamovi.org. This software provides a range of statistical analysis tools equivalent to proprietary alternatives like IBM SPSS. Jamovi is available under the GNU General Public License (GPL) and is freely accessible for download and use.
