# Factors Associated with Progressive Liver Disease in Untreated HBV patients in Tunisia

**DOI:** 10.12688/f1000research.157075.2

**Published:** 2025-05-19

**Authors:** Sana Rouis, soumaya mrabet, Mohamed Ferjaoui, Nedia Ben Lasfar, Jihene Sahli, Syrine Boujamline, Rym Ayari, Maha Abid, Manel Ben Selma, Mariem Ben Ticha, Foued Bellazreg, Elhem Ben Jezia, Amel Letaief, Wissem Hachfi

**Affiliations:** 1 Department of Infectious Diseases, Faculty of Medicine of Sousse, University of Sousse, Ibn El Jazzar University Hospital, Kairouan, Tunisia; 2 Department of Hepato-gastroenterology, Faculty of Medicine of Sousse, University of Sousse, Farhat Hached University Hospital, Tunisia; 3 Department of Infectious Diseases, Faculty of Medicine of Sousse, University of Sousse, Farhat Hached University Hospital, Tunisia; 4 Department of Familial and Community Medicine, Faculty of Medicine of Sousse, University of Sousse, Sousse, Tunisia; 5 Faculty of Medicine of Sousse, University of Sousse, 4000, Sousse, Tunisia

**Keywords:** hepatitis b virus, indeterminate phase, liver fibrosis, risk factors

## Abstract

**Background:**

Antiviral therapy is not routinely recommended for chronic hepatitis B virus (HBV) infection in patients exhibiting persistently elevated serum HBV DNA levels (>2000 IU/mL), normal alanine aminotransferase (ALT), and without significant liver fibrosis – a clinical state termed the indeterminate phase. Recognizing the evolving treatment landscape, this study aimed to identify factors associated with liver fibrosis progression in chronic HBV patients within this indeterminate phase.

**Methods:**

This retrospective longitudinal cohort study was conducted in the Infectious Disease and Hepato-gastroenterology departments of Farhat Hached University Hospital in Sousse, Tunisia, between January 2008 and January 2022. We included HBsAg-positive patients initially untreated, presenting at evaluation with a viral load >2,000 IU/mL for at least six months, normal ALT (<40 IU/L), and a fibrosis score of F0 and/or F1 (determined by liver biopsy or FibroScan). Univariate and logistic regression analyses were performed to identify factors associated with liver fibrosis progression.

**Results:**

A total of 97 patients were included, with a median age of 32.9 ± 9.1 years, and a female predominance (M/F ratio = 0.64). Fibrosis progression was observed in 16 patients (16.5%), with a mean delay of 70.9 ± 41.1 months. Univariate analysis revealed associations between fibrosis progression and the presence of comorbidities (p=0.001), a high initial viral load (p=0.004), elevated liver enzymes (p=0.001), and an increase in viral load during follow-up (p=0.002). Multivariate analysis identified comorbidities (p<0.001) and changes in ALT levels (p<0.001) as independent predictors of fibrosis progression. The AUROC of the initial viral load was 0.664 (95%CI: 0.500-0.820).

**Conclusion:**

The presence of comorbidities and changes in ALT levels during follow-up were associated with fibrosis progression in the indeterminate phase of chronic HBV infection. These findings suggest a potential rationale for extending therapeutic indications to include this patient population.

## Introduction

Infection with hepatitis B virus (HBV) is a public health problem with significant morbidity and mortality associated with cirrhosis and its complications. ^1^ As per the 2017 WHO global report, over two billion individuals have been exposed to HBV ^2^ while the 2024 WHO global hepatitis report indicates a chronic hepatitis B prevalence of 2.1% in the Eastern Mediterranean Region. ^1^ In North Africa, HBV infection has been described as a major etiological agent for the development of hepatocellular carcinoma (HCC). ^3^ In Tunisia, the national prevalence of HBs Ag 1.7%. ^4^

Chronic HBV infection is a dynamic process that reflects the interaction between HBV replication and host immune response. According to the well-established guidelines for HBV monitoring, the decision to initiate antiviral therapy with nucleotide/nucleoside analogues in chronic hepatitis B (CHB) is primarily based on the combination of the following three criteria: viral load of HBV, ALT levels, and the extent and severity of liver histopathological changes, as assessed using standardized scoring systems such as the METAVIR, Ishak, or NAS (NAFLD Activity Score) systems. ^5^ Thus, antiviral therapy with nucleotide/nucleoside analogues is indicated only in patients with a viral load> 2,000 IU/mL, whether or not associated with elevated liver enzymes and with significant hepatic fibrosis (≥F2) on liver biopsy or elastography. However, in the presence of a viral load> 2000 IU/mL, there is a risk of disease progression to cirrhosis and HCC. ^5^ Untreated patients should be monitored by regular liver enzymes and HBV viral load determinations, as well as by noninvasive fibrosis assessment and liver ultrasound. However, the modalities of monitoring its rhythm are not well established, and there are few data concerning the natural history of these patients. ^5^

Patients with a viral load >2,000 IU/mL, but who do not meet the criteria defining therapeutic indications, have recently been classified in the so-called “indeterminate phase”. ^6– 8^ They present an increased risk of progression of hepatic fibrosis, which has been objectified on the basis of noninvasive markers of fibrosis, such as the FIB-4 Score. This progression was on the order of 11% per year in the absence of certain factors, such as advanced age, metabolic syndrome, and chronic alcohol intake. ^9^

The virosuppression achieved by anti-viral therapy in CHB reduces the risk of progression to fibrosis and HCC. Antiviral therapy with nucleotide/nucleoside analogues has been shown to prevent around 60% of new cases of HCC over a 10-year period. ^10^

However, other studies have shown that the risk of fibrosis progression, cirrhosis, and its complications is minimal and comparable between treated and untreated patients. ^11– 13^

In this study, we aimed to identify the factors associated with fibrosis progression in patients with chronic HBV infection in the indeterminate phase.

## Methods

### Study design

This study, designed as a retrospective longitudinal cohort, was conducted within the Infectious Diseases and Hepato-Gastroenterology departments at Farhat Hached University Hospital in Sousse, Tunisia—a facility located within the WHO Eastern Mediterranean Region. The study spanned a period of 14 years, from January 2008 to January 2022.

### Study setting and participants

We included all adult patients with chronic HBV infection in the “indeterminate phase” with HBsAg positive not initially treated, presenting at the time of initial evaluation a viral load> 2,000 IU/mL for at least six months, normal ALT (< 40 IU/L), and fibrosis score F0 and/or F1 (on liver biopsy or FibroScan).

We did not include patients with cirrhosis, co-infection with hepatitis D virus (HDV), hepatitis C virus (HCV), or human immunodeficiency virus (HIV), a personal or family history of HCC, patients treated at the time of diagnosis, immunotolerant patients, and those receiving pre-emptive treatment during follow-up.

Patients with a positive HBsAg are typically referred by primary care physicians following screening tests conducted prior to blood transfusions, during pre-employment checkups, or as part of prenatal care for pregnant women. These patients are usually monitored under the national health insurance system, either through a public healthcare plan or a reduced-cost scheme, ensuring access to necessary medical follow-up and treatment. This approach helps in managing the condition effectively while providing financial support to those in need.

### Data analysis

Data were collected from the medical records of patients using a standardized data form. In fact, data collection began at the start of the cohort in January 2008. Data processing began in May 2023, when the team started the study. The baseline data comprised sociodemographic details, including age, sex, profession, and address. We also considered comorbidities such as arterial hypertension, (which is diagnosed based on ≥2 elevated blood pressure readings on separate occasions, with categories including normal (<120/80 mmHg), elevated (120–129/<80 mmHg), stage 1 (130–139/80–89 mmHg), and stage 2 (≥140/≥90 mmHg) hypertension, ^14^ dyslipidemia (diagnosed through a fasting lipid profile, which measures total cholesterol, LDL-C (the primary marker for atherosclerosis), HDL-C, and triglycerides. Elevated LDL-C is categorized as optimal (<100 mg/dL), borderline high (130–159 mg/dL), or high (≥160 mg/dL). Low HDL-C is defined as <40 mg/dL in men and <50 mg/dL in women, while hypertriglyceridemia is considered normal (<150 mg/dL) or high (≥200 mg/dL), ^15^ diabetes mellitus (a chronic metabolic disorder characterized by high blood sugar levels due to either insufficient insulin production, ineffective insulin action, or both), ^16^ chronic alcohol intake (two to three standard glasses per day, a “standard glass,” is a measure used to quantify alcohol consumption uniformly. It varies slightly by country, but generally, a standard glass contains about 10 grams of pure alcohol, ^17^ body mass index (classified into 4 categories: <25 kg/m ^2^/25-30/30-35/ >35 kg/m ^2^), hepatic steatosis (on abdominal ultrasound and/or liver biopsy), HBe Ag (positive or negative), initial ALT level (normal is defined as < 40 IU/L), and baseline viral load (over the course of the disease, classified into 2 categories:moderate (2000–20,000 IU/mL) and high (>20,000 IU/mL).

At the Farhat Hached Laboratory, hepatitis B virus (HBV) testing is performed using **ELISA-based assays** for detecting HbsAg, anti-HBs, and anti-HBc antibodies. Viral load quantification (HBV DNA) is conducted via **real-time PCR**. The microbiology laboratory often considers a viral load below 20 IU/mL as undetectable or very low, which is a good indicator of infection control. Liver enzymes (ALT/AST) are measured using **automated biochemistry analyzers** with standardized reagents. Positivity thresholds for HBV serology follow manufacturer guidelines (e.g., HbsAg ≥ 0.05 IU/mL), while liver enzymes report in **IU/L** with elevated thresholds (ALT > 40 IU/L for males, > 35 IU/L for females **)**. Results are interpreted per WHO/local guidelines, ensuring traceability and reproducibility.

Liver biopsy in the context of HBV infection is a critical diagnostic tool used to assess the extent of liver damage, fibrosis, and inflammation. The inclusion criteria for performing a liver biopsy typically include patients with chronic HBV infection who exhibit elevated liver enzymes persistently above the upper limit of normal, or those with indeterminate or conflicting results from imaging and serological tests. ^5^ Additionally, patients who are being considered for antiviral therapy, especially when the decision is not clear-cut based on non-invasive methods, may also be included. Exclusion criteria generally involve patients with contraindications to biopsy, such as severe coagulopathy, thrombocytopenia, or ascites, as these conditions increase the risk of complications like bleeding. Patients with acute HBV infection or those who have already been diagnosed with advanced cirrhosis and for whom biopsy would not alter management are also typically excluded. ^5^ The decision to proceed with a liver biopsy should always be individualized, weighing the potential benefits against the risks, and should be discussed thoroughly with the patient.

We specify that the FIB-4 and APRI scores were not used in this study, as these are non-invasive scores that emerged in 2014, ^18^ while our cohort began in 2008. Therefore, we could not integrate these scores for former patients.

During the follow-up, the viral load was monitored (increasing/decreasing/fluctuating), ALT rate (/6 months) (normal/high/fluctuating), METAVIR score at liver biopsy control, and liver stiffness at elastography (FibroScan) control. The word “fluctuating” refers to variation in the amount of hepatitis B virus (HBV) DNA or ALT value detected in a patient’s blood over time. The following events were noted: progression of fibrosis, cirrhosis, HCC, HBs or Hbe seroclearance (to look for loss of HBs or Hbe Ag), HBs, or Hbe seroconversion (appearance of Anti HBs or Anti Hbe antibodies).

The indications for antiviral therapy with nucleotide/nucleoside analogues during follow-up included the progression of fibrosis (defined by an increase of at least 1 point in METAVIR score on liver biopsy, ^1^ or an increase in liver stiffness of at least 1 kPa on FibroScan), progression to cirrhosis (defined by a METAVIR F4 score on FibroScan, or an elasticity greater than 15 kPa on FibroScan and/or the presence of indirect clinical, biological, and morphological signs), the occurrence of HCC (for which diagnosis is based on hepatic angioscan or MRI data, or on histological data from liver nodule biopsy).

During the study period, the **EASL guidelines** were primarily referenced, as they were the most up-to-date recommendations at the time. These guidelines emphasized the use of **nucleos(t) ide analogues (nAs)** such as **entecavir (ETV)**, or **lamivudine (3TC)** as first-line antiviral therapies for chronic HBV infection. **Pegylated interferon-alpha (PEG-IFN)** was also considered in select cases, particularly for patients with HBeAg-positive disease, having a co-infection VHD, or those seeking a finite treatment duration. ^5^ The decision to initiate treatment was based on criteria such as elevated HBV DNA levels, significant liver inflammation or fibrosis, and elevated ALT levels, in alignment with the recommendations. Thus, the treatment approach adhered to the EASL guidelines, ensuring evidence-based and standardized care for HBV-infected patients. ^5^

### Statistical analysis

Statistical analysis was performed using IBM SPSS 20.0 software ( https://www.ibm.com/fr-fr/products/spss-statistics ).

Quantitative variables are expressed as the mean ± SD. Qualitative variables are expressed as percentages. The comparison of two means on independent series was carried out using Student’s t-test. The comparison of percentages was performed using the Pearson χ ^2^ test and, in the event of non-validity, using the exact two-sided Fisher test. Associations between the different variables and liver fibrosis progression were estimated in a univariate analysis and a logistic regression analysis with the expression of an Odds Ratio (OR) with a 95% confidence interval. The relevant factors were analyzed, and their diagnostic value was evaluated using the receiver operating characteristic (ROC) curve and the area under the ROC curve (AUROC). For all statistical tests, statistical significance was set at p<0.05.

## Results

### Enrolled patients

**Figure 1.**
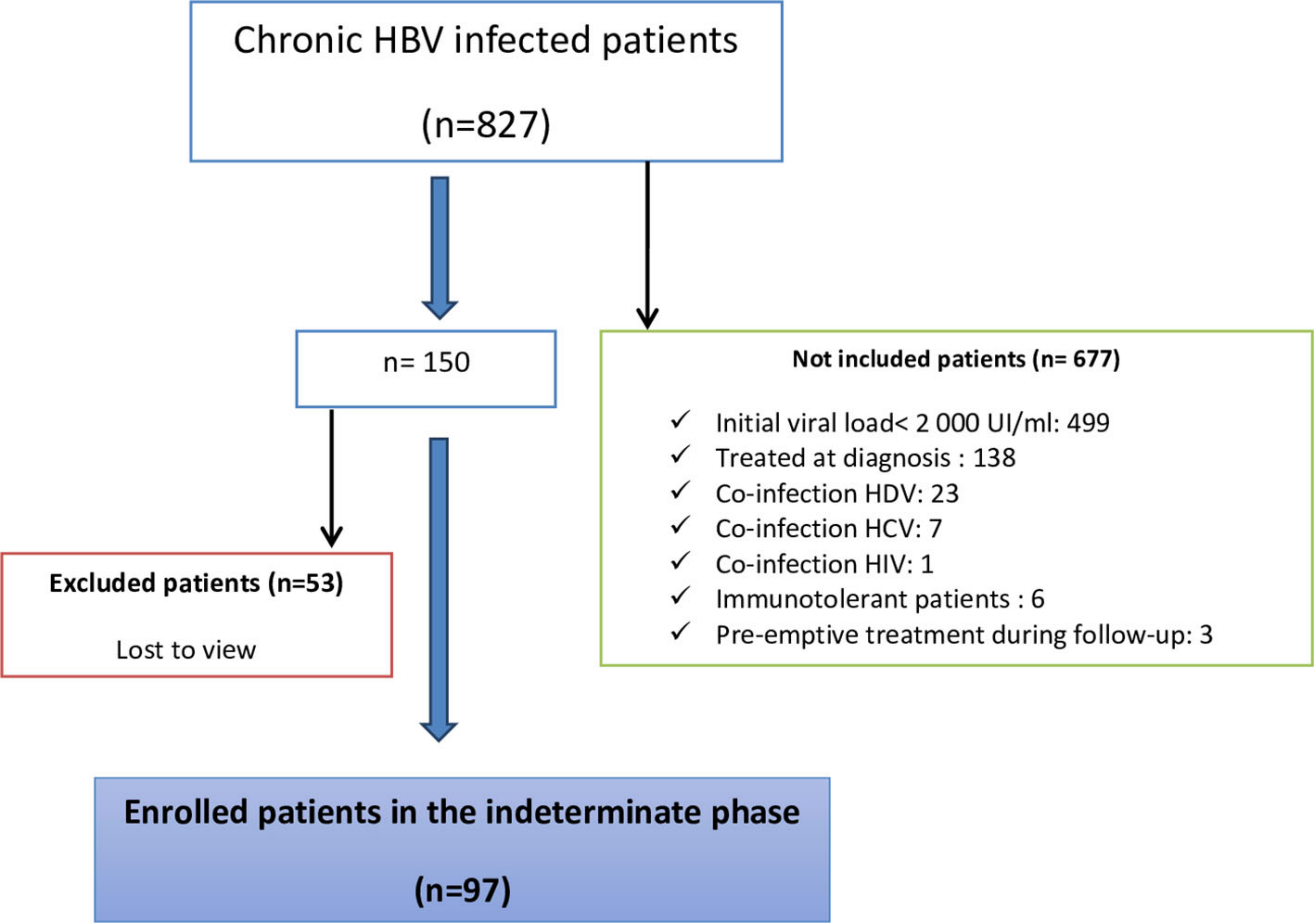
The flow chart of the study population.

A total of 827 patients were followed up for chronic HBV infection. There were 53 patients who were excluded because they were lost to follow-up. The flow diagram of the study population is shown in Figure 1. Moreover, 677 patients were not included in the study; among them, 499 had a baseline viral load of < 2000 IU/mL. Finally, 97 patients who fulfilled the inclusion criteria were included ( Figure 1).

### Baseline characteristics

The baseline characteristics of the included patients are shown in Table 1. The mean age of the participants was 32.9 ± 9.1 years. The mean initial viral load was 8 182 122.4 IU/mL. Among these patients, 38 (39.2%) were men (sex ratio M/F=0.64) and 95 (97.9%) were HBeAg-negative.

**Table 1.** Baseline Characteristics of enrolled patients with chronic HBV infection in the indeterminate phase (n = 97).

Variables		n (%)
**Age, mean±SD (years**)	32.9±9.1	
<20		6 (6.2)
20-40		72 (74.2)
40-60		18 (18.6)
>60		0 (0.0)
**Gender, n (%)**		
Male		38 (39.2)
Female		59 (60.8)
**Underlying comorbidities, n (%)**		13 (13.4)
Dyslipidemia		7 (7.2)
Diabetes mellitus		4 (4.1)
High blood pressure		2 (2.1)
**BMI, mean±SD (kg/m** ^**2**^ **)**	26.1±4.6	
<25		30 (30.9)
25-30		32 (33.0)
30-35		9 (9.3)
>35		2 (2.1)
**HbeAg, n (%)** Positive		2 (2.1)
Negative		95 (97.9)
**Baseline viral load, mean±SD (UI/l)**	8 182 122.4±31488242.1	
Moderate (2000-20000 cp/mL), n (%)		81 (83.5)
High (> 20000 cp/mL), n (%)		16 (16.5)
**Baseline fibrosis, n (%)**		62 (60.2)
**Tests performed to assess fibrosis, n (%)**		
Liver biopsy		40 (41.2)
FibroScan		57 (58.8)

Underlying comorbidities were observed in 13.4% of patients (n=13). These comorbidities included dyslipidemia (n=7), diabetes (n=4), and high blood pressure (n=2). The mean Body Mass Index (BMI) was 26,1± 4.6 kg/m ^2^. The initial assessment of fibrosis was performed in all patients (liver biopsy (41.2%) and elastography (58.8%)) ( Table 1). The mean initial liver stiffness was 4,9± 1.2 kPa.

### Outcomes

The median follow-up period was 105.2 ± 48.4 months. Fibrosis progression was noted in 16 patients (16.5%), with an average time to fibrosis progression of 70.9 ± 41.1 months. Antiviral therapy with nucleotide/nucleoside analogues was initiated. Complications that occurred in the enrolled patients included cirrhosis (n=3), HCC (n=1), and death (n=1). Serologically, 11 patients (10.7%) had a loss of HBsAg and five had a loss of HBe Ag (4.9%), with a mean delay of 87± 49 and 51± 24.7 months ( Table 2).

**Table 2.** Outcomes of enrolled patients with chronic HBV infection in the indeterminate phase (n = 97).

Variables		n (%)
Changes in ALT, n (%)	Normal	86 (88.7)
	Fluctuating elevated liver enzymes	10 (10.3)
	Persistent elevated liver enzymes	1 (1.0)
Viral load during follow-up, n (%)	Low (<2000 cp/mL)	33 (34.0)
	Moderate (2000-20000 cp/mL)	10 (10.3)
	High (>20000 cp/mL)	7 (7.2)
	Flucuating	47 (48.5)
Direction of evolution of viral load, n (%)	Stable	8 (8.2)
	Increasing	8 (8.2)
	Decreasing	26 (26.8)
	Fluctuating	55 (56.7)
Liver Fibrosis, n (%)	Stable	81 (83.5)
	Progression	16 (16.5)
	Regression	0 (0.0)
Use of antiviral treatment during follow-up, n (%)		16 (16.5)
Changes in serological profile, n (%)	Loss of HBs Ag	10 (10.3)
	Seroconversion HBs	8 (8.2)
	Loss of HBe Ag	2 (2.1)
	Seroconversion HBe	2 (2.1)
Complications, n (%)	Cirrhosis	3 (3.1)
	HCC	1 (1.0)
	Death	1 (1.0)

### Risk factors associated with liver fibrosis

**Figure 2.**
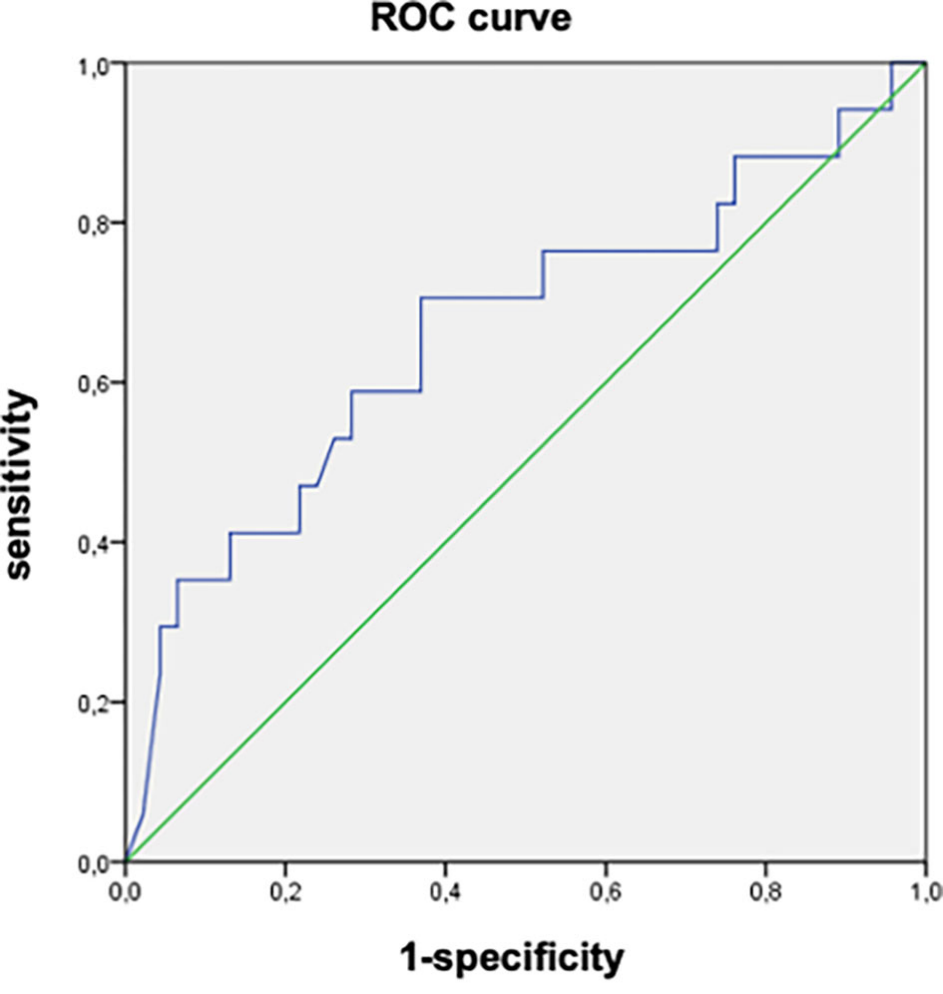
AUROC of the initial viral load as a factor associated with liver fibrosis progression in patients with chronic HBV infection in the indeterminate phase. (AUROC = 0.664 (95% CI: 0.500-0.820), Cut-off = 8090 IU/L, sensbility = 70.3% and specificity = 63%).

In the univariate analysis, factors associated with the progression of fibrosis were the presence of comorbidities (p=0.001), high initial viral load (p=0.004), appearance of elevated liver enzymes (p=0.001), and increased viral load (p=0.002) during follow-up ( Table 3). The AUROC of the initial viral load was 0.664 (95%CI: 0.500-0.820). An initial viral load of 8090 IU/mL was associated with the progression of fibrosis with a sensitivity of 70.3% and specificity of 63% ( Figure 2).

**Table 3.** Factors associated with progression of fibrosis in patients with chronic HBV infection in the indeterminate phase in the univariate analysis (n=97).

Factors	Progress of liver fibrosis	No progression of liver fibrosis	p
	N=16	N=81	
**Age, mean (years)**	33.1	31.7	0.708
<20 years old	1 (16.7)	5 (83.3)	0.799
20-40 years old	11 (15.3)	61 (84.7)	
40-60 years old	4 (22.2)	14 (77.8)	
>60 years old	0 (0.0)	0 (0.0)	
**Sex, n (%)**			
Male	6 (15.8)	32 (84.2)	0.881
Female	10 (16.9)	49 (83.1)	
**Smoking, n (%)** Yes	1 (14.3)	6 (85.7)	0.929
No	5 (15.6)	27 (84.4)	
**Alcohol intake, n (%)** Yes	2 (25.0)	6 (75.0)	0.486
No	13 (15.5)	71 (84.5)	
**Comorbidities, n (%)** Yes	8 (61.5)	5 (38.5)	**0.001**
No	8 (9.5)	76 (90.5)	
**BMI, mean (kg/m ^2^)**	24,3	26,1	0.411
**Steatosis, n (%)** Yes	4 (16.7)	20 (83.3)	0.979
No	12 (16.4)	61 (83.6)	
**Baseline absolute ALT value, n (%)**			
≤40 IU/L	15 (16.1)	78 (83.9)	0.640
>40 IU/L	1 (25.0)	3 (75.0)	
**HBe Ag, n (%)**			
Positive	1 (50.0)	1 (50.0)	0.197
Negative	15 (15.8)	80 (84.2)	
**Baseline viral load, n (%)**			
Moderate (2000-20000 cp/mL)	9 (11.1)	72 (88.9)	**0.004**
High (>20000 cp/mL)	7 (43.8)	9 (56.2)	
**Changes in ALT, n (%)**			
Normal	9 (10.5)	77 (89.5)	**0.001**
Persistant elevated liver enzymes	0 (0.0)	1 (100.0)	
Fluctuating elevated liver enzymes	7 (70.0)	3 (30.0)	
**Changes of viral load, n (%)**			
Low (<2000 cp/mL)	0 (0.0)	33 (100.0)	
Moderate (2000-20000 cp/mL)	5 (50.0)	5 (50.0)	**0.001**
High (>20000 cp/mL)	4 (57.1)	3 (42.9)	
Fluctuating	7 (14.9)	40 (85.1)	
**Direction of evolution of viral load, n (%)**			
Stable	0 (0.0)	8 (100.0)	
Increasing	5 (62.5)	3 (37.5)	**0.002**
Decreasing	2 (7.7)	24 (92.3)	
Fluctuating	9 (16.4)	46 (83.6)	

In the multivariate analysis, the two independent predictors of fibrosis progression were the presence of comorbidities (Odds Ratio (95% CI)=53.345 (8.612-330.437), p<0.001) and the fluctuating/elevation in ALT levels (Odds Ratio (95% CI)=8.539 (3.168-23.018), p<0.001). In our study population, the baseline viral load was not found to be an independent predictor of fibrosis progression ( Table 4).

**Table 4.** Factors associated with progression of fibrosis in patients with chronic HBV infection in the indeterminate phase in the logistic regression analysis (n=97).

Factors	Odds Ratio	(95% CI)	p
**Comorbidities, n (%)** Yes	53.345	[8.612-330.437]	**<0.001**
No		**Ref**	
**Changes in ALT, n (%)**			
Normal			
Persistant elevated liver enzymes	8.539	**Ref**	
Fluctuating elevated liver enzymes		[3.168-23018]	**<0.001**

## Discussion

The main findings of our study were as follows: First, progression of fibrosis was observed in 16 patients (16.5%), with a mean delay of 70.9±41.1 months. Second, the factors associated with the progression of fibrosis were the presence of comorbidities, high initial viral load, elevated liver enzymes, and increased viral load during follow-up. An initial viral load of 8090 IU/mL was associated with the progression of fibrosis, with a sensitivity of 70.3% and specificity of 63%.

While this study was conducted prior to the release of the 2024 WHO guidelines ^19^ which now recommend antiviral therapy for a broader range of patients with chronic HBV, our findings regarding the factors associated with disease progression in untreated individuals remain relevant for understanding the natural history of the disease in populations where treatment access may be limited or where patients do not meet current treatment criteria.

### Frequency of the indeterminate phase among chronic HBV-infected patients

Our study included chronic HBV infected patients in the indeterminate phase, which represented 11.7 % of all the patients belonging to the CHB cohort of Farhat Hached University Hospital (n=827). Although the treatment guidelines for chronic HBV infection are well-defined, greater focus should be given to individuals who do not meet these criteria, particularly those classified in the “indeterminate phase,” as this phase is not always benign.

In our study, these patients represented 11.7% of all patients. This finding was less than that reported in a retrospective multicenter cohort study conducted in the USA and Taiwan China, ^6^ in 3366 CHB patients were followed up for at least 1 year.

The findings showed that patients in the indeterminate phase accounted for, on average, 31.8% of the Chinese and Taiwanese cohorts and 38.7% of the American cohort. Additionally, there were 4759 CHB patients in Nanjing, China, of which 27.8% were in the indeterminate phase, according to Yao et al. ^8^ The percentage of patients in the indeterminate phase found in our study may be explained byour younger population with a predominance of inactive carriers among all the HBV-infected patients followed in our center.

### Factors associated with liver fibrosis progression

Jiang et al. reported that 24.3% of patients in the indeterminate phase are at risk of disease progression. ^20^ In our study, among the 97 patients, 16 (16.5%) developed fibrosis, leading to the initiation of antiviral therapy with nucleotide/nucleoside analogues. In a previous study that included 234 patients with CHB who did not meet the treatment criteria at presentation and during a median follow-up period of 51 months, 19.2% of patients transitioned to a more active disease phase and 18.8% started antiviral therapy. ^21^ Huang et al. reported that among 1303 patients in the indeterminate phase, 283 (21.7%) transitioned to immune-active disease by up to 10 years of follow-up evaluation. ^6^

In our study, in the univariate analysis, factors associated with the progression of fibrosis were the presence of comorbidities, a high baseline viral load, the development of elevated liver enzymes, and the increase in viral load during follow-up. In logistic regression analysis, the independent predictive factors of liver fibrosis progression were the presence of comorbidities and changes in ALT levels.

A review of the literature revealed only one study that examined the factors associated with fibrosis in patients in the indeterminate phase of the disease. In this retrospective cohort study involving 634 patients with CHB infection in the indeterminate phase, ^20^ the authors found that the statistically significant variables that could affect liver fibrosis were a low/moderate HBV DNA level at the initial assessment and an increased gamma-glutamyl transpeptidase (GGT) level. In contrast, increased aspartate transaminase to platelet ratio index (APRI) and (liver inflammation and fibrosis 5) LIF-5 values ^22^ were independent risk factors for liver fibrosis in the indeterminate phase. ^20^ This study showed that regardless of ALT values, patients with an initial low/moderate viral load had more severe liver disease, in contrast to our findings. In fact, a high viral load leads to dysfunction of HBsAg-specific cytotoxic T lymphocytes, resulting in immune tolerance, which is characterized by high viral replication, normal liver function, minimal immune response, and HBeAg positivity, often lasting years with low risk of liver damage but requiring monitoring for potential progression. ^5^ However, during prolonged reproduction, HBV interacts with the host immune system and induces cumulative immune damage and, consequently, liver damage. ^20^ No other studies have analyzed comorbidities as an associated factor with the progression of fibrosis or other evolutionary parameters, such as the development of elevated liver enzymes in patients in the indeterminate phase. In fact, patients with comorbidities are not often included in studies evaluating hepatic fibrosis in HBsAg-positive patients because the progression of fibrosis may be linked to metabolic dysfunction-associated liver disease. In our research, although there is a significant link between comorbidities and fibrosis, additional prospective studies are necessary.

On the other hand, we found that an initial viral load of 8090 IU/mL was associated with the progression of fibrosis, with an AUROC of 0.664 (95%CI: 0.500-0.820), a sensitivity of 70.3%, and a specificity of 63%. In the same study by Jiang et al., ^20^ low/moderate viral load was an independent factor for liver fibrosis, with an AUROC of 0.799 (95%CI: 0.760–0.838) without defining a specific cut-off. Chen et al. showed that serum HBV DNA levels in patients in the indeterminate phase were significantly higher in those with advanced inflammation and fibrosis. ^7^ Elevated serum HBV DNA levels are a risk factor for significant liver inflammation in patients with CHB, which is consistent with the findings of other studies. ^23, 24^ While we suggest a threshold viral load of 8090 IU/mL to inform treatment decisions for indeterminate patients, additional research is necessary to establish the viral load cut-off linked to a significant risk of fibrosis progression in patients during the indeterminate phase.

### Occurrence of HCC and cirrhosis

In the present study, only one patient developed HCC (1%) and 26 (2%) in Hunag’s study. ^6^ In fact, the correlation between viral load and the progression of end-stage liver disease (such as HCC) remains controversial. In a recent meta-analysis, the pooled annual HCC incidence was 2.54 cases per 1.000 person years (95% CI, 1.14–4) for patients in the indeterminate phase. ^25^

According to Huang et al., In addition to age 45 years and older (aHR, 20.8; 95% CI, 2.8–156.7; p=0.003), the indetermined period was independently linked to a higher risk of HCC development (aHR, 14.1; 95% CI, 1.3–153.3; p=0.03) than the inactive phase. ^6^ Contrary to the findings reported by Lee et al., in which the authors assessed the untreated persistently elevated serum HBV patient group (patients in the indeterminate phase) and analyzed the cumulative HCC risk at 3, 5, 7, and 9 years (n = 67), which were 0%, 0%, 2.9%, and 2.9%, respectively. ^12^

In contrast, we found that three patients developed cirrhosis (3.1%). In a study by Yapali et al., which included 234 patients who did not meet the criteria for antiviral therapy with nucleotide/nucleoside analogues at presentation, none of the patients experienced cirrhosis during the follow-up. ^21^ These results are in contrast to those of Huang et al., who found a higher 10-year cumulative incidence of cirrhosis among indeterminate patients who remained indeterminate versus inactive patients who remained inactive, 8.8% (95% CI, 6.5–11.8) vs 3.5% (95% CI, 2.5–5.0; p<.0001). ^6^

In our study, the low incidence of HCC and cirrhosis is anticipated, largely due to the young average age of the cohort, and this stands in contrast to the higher complication rates observed in older or Asian cohorts.

### Antiviral therapy with nucleotide/nucleoside analogues in patients with indeterminate phase

Antiviral therapy with nucleotide/nucleoside analogues indications are generally provided to individuals at a high risk of disease progression, namely those with elevated ALT levels, active viral replication, and advanced fibrosis or cirrhosis. ^5^

As reported above, Huang et al. found that, without treatment, 21.7% of patients in the indeterminate phase had fibrosis progression and became immune active. These patients had a higher 10-year cumulative incidence of cirrhosis than those in the inactive phase and a 14 times higher risk of HCC development. ^6^ Similarly, another observational study including 5414 patients, demonstrated that, compared to patients receiving oral antiviral therapy in the active phase, untreated HBeAg-negative CHB patients in the indeterminate phase had a considerably greater risk of HCC and mortality. ^26^ As long as HBV DNA is found, several experts have suggested that therapy should start as soon as feasible to lower the chance of the disease progressing. ^27^ Therefore, Zhou et al. recommended that antiviral therapy should be initiated in HBeAg-negative patients with normal ALT and HBV DNA ≥ 2 000 IU/mL. ^27^ Based on our findings, we propose to treat patients with chronic HBV infection in the indeterminate phase from a viral load value of 8090 IU/mL, with underlying comorbidities and developing elevated liver enzymes during flollow-up. However, the clinical benefits of antiviral therapy in this population need to be confirmed in future studies.

### Limitations

To our knowledge, this is the first large-scale cross-sectional study of Tunisian patients with chronic HBV infection in the indeterminate phase. However, our study has some limitations. First, many patients were lost to follow-up and were excluded from the final analysis. Second, this study did not determine the HBV genotypes. The dominant genotype of HBV in Tunisia is genotype D, ^28^ and it has been demonstrated that genotype C infections are more prone to progress to HCC earlier, which goes some way explains the low frequency of HCC in our patients. ^24^ On the other hand, the study acknowledges the limitations posed by the small sample size and the resulting wide confidence intervals, which may affect the precision and generalizability of the findings. The rationale for collecting data on specific comorbidities was driven by their established relevance to the research question and their potential to influence outcomes, while other conditions were excluded to maintain focus and avoid diluting the analysis. Ultimately, the findings of our study provide a groundwork for future, more extensive research that can expand on these initial results.

## Conclusions

In summary, fibrosis progression occurred in 16.5% of the patients with chronic HBV infection in the indeterminate phase. The main risk factors associated with liver fibrosis were the presence of comorbidities, high initial viral load with a cut-off of 8090 IU/mL, the appearance of elevated liver enzymes, and an increase in viral load during follow-up. Further studies are required to determine whether early antiviral therapy with nucleotide/nucleoside analogues can reduce the incidence of cirrhosis and HCC in these patients.

## Ethics and consent

This study was conducted in accordance with the standards of ethics of the research. Anonymity and data confidentiality were guaranteed for all patients and written informed consent for participation in the study was obtained. At the time of data collection, the study was designed as a retrospective review of existing patient records, which did not initially anticipate publication or require additional interventions beyond routine clinical care. Consequently, an ethical approval was not sought prospectively. However, prior to manuscript submission for several months, we obtained ethical clearance retrospectively from the Ethical Committee of the Faculty of Medicine of Sousse, Tunisia on January 10, 2024, which reviewed and approved the use of the data for research purposes [Ethical Committee Number AVIS Number 220 (Ref: CEFMS 220/2024)].

## Data Availability

The project contains the following underlying data:
• [Figshare]: sana rouis (2024). [Factors associated with progression of fibrosis in chronic hepatitis B virus infection in the indeterminate phase]. figshare. https://doi.org/10.6084/m9.figshare.28007054 Data_VHB. Dataset. ^29^ • [Figshare]: sana rouis (2024).[Factors associated with progression of fibrosis in chronic hepatitis B virus infection in the indeterminate phase] (data collection sheet). https://doi.org/10.6084/m9.figshare.27172914.v1. ^30^
• [Figshare]: sana rouis (2024). [Factors associated with progression of fibrosis in chronic hepatitis B virus infection in the indeterminate phase] (patient consent form). https://doi.org/10.6084/m9.figshare.27172956.v1. ^31^
•
[Figshare]: sana rouis (2024) [Factors associated with progression of fibrosis in chronic hepatitis B virus infection in the indeterminate phase] (patient information letter). https://doi.org/10.6084/m9.figshare.27902625.v1. ^32^
• [Figshare]: sana rouis (2024) [Factors associated with progression of fibrosis in chronic hepatitis B virus infection in the indeterminate phase] (STROBE statement). https://doi.org/10.6084/m9.figshare.29064221.v1. ^33^ Data are available under the terms of the Creative Commons Attribution 4.0 International license (CC-BY 4.0). Our article answers the STROBE checklist for observational studies (Extended data).

## Software availability statement

The data analysis was conducted using Jamovi version 2.3, which is open-access software available at https://www.jamovi.org. This software provides a range of statistical analysis tools equivalent to proprietary alternatives like IBM SPSS. Jamovi is available under the GNU General Public License (GPL) and is freely accessible for download and use.
