# Supplementary material for: Factors Associated with Progressive Liver Disease in Untreated HBV Patients in Tunisia
Source: F1000Res. 2025 Aug 27;14:11. Originally published 2025 Jan 2. [Version 3] doi: 10.12688/f1000research.157075.3 (PMC12423619; doi:10.12688/f1000research.157075.3)
Supplement: Peer review history

# Reviewer report: Reviewer response for version 3

**DOI:** 10.5256/f1000research.187074.r432145

**Published:** 2025-12-11

**Authors:** Tamer A. Addissouky

**Affiliations:** 1 Menoufia University, Shebeen El-Kom, Menofia Governorate, Egypt

**Manuscript Title:** Factors Associated with Progressive Liver Disease in Untreated HBV Patients in Tunisia

**Journal:** F1000Research

**Manuscript ID:** f1000research.157075.v3

**Summary of Revisions Made**

- **Title and Abstract:**
  The authors have clarified the geographic context by clearly specifying Tunisia and the WHO Eastern Mediterranean Region. The abstract now accurately reflects the new WHO 2024 guidelines, acknowledging the obsolescence of the “indeterminate phase” terminology and highlighting how the study supports expanded treatment indications.
- **Introduction:**
  Improved logical flow and clarity with defined terminology and updated epidemiological data from the 2024 WHO report. The rationale for the study is now clearly articulated, reflecting the evolving HBV treatment landscape.
- **Methods:**
  The study design is explicitly described as a retrospective longitudinal cohort. Detailed information on patient recruitment, laboratory methods, viral load standardization, and treatment guidelines during the study period has been added. Handling of missing data and confounding factors is explained. The STROBE checklist is provided as supplementary material.
- **Results:**
  Viral load data are reported as median and interquartile range (IQR) to avoid skewness by outliers. Viral load units standardized to IU/mL. Clarifications on timing of antiviral therapy initiation and detailed fibrosis progression outcomes are included. Tables and figures have been corrected for consistency, clarity, and language.
- **Discussion:**
  The authors discuss their findings within the context of current literature and 2024 WHO guidelines. The importance of comorbidities as independent predictors of fibrosis progression is emphasized, with appropriate caution regarding causality and a call for prospective studies. The potential confounding effect of metabolic-associated fatty liver disease (MAFLD) is discussed.
- **Language and Terminology:**
  Person-centered language has been adopted throughout. Typographical errors and inconsistencies have been corrected. Technical terms have been standardized and clarified for broader accessibility.
- **Limitations:**
  The modest sample size and resulting wide confidence intervals are acknowledged with appropriate caution on the generalizability of findings. The exclusion of HBV genotyping is noted, with some discussion on genotype distribution relevant to the Tunisian population.
- **Ethics and Data Availability:**
  Ethical approval has been confirmed retrospectively. Complete datasets and supporting materials are openly accessible under a CC-BY 4.0 license.

**Acceptance Recommendation**

 After thorough revision addressing all reviewer comments, the manuscript now meets the journal’s standards for clarity, methodological rigor, and relevance. It provides valuable longitudinal data on fibrosis progression among untreated HBV patients in a region with limited prior data and supports the rationale behind evolving global treatment guidelines.

**I recommend acceptance for Indexing.**

Is the work clearly and accurately presented and does it cite the current literature?

Yes

If applicable, is the statistical analysis and its interpretation appropriate?

Yes

Are all the source data underlying the results available to ensure full reproducibility?

Yes

Is the study design appropriate and is the work technically sound?

Yes

Are the conclusions drawn adequately supported by the results?

Yes

Are sufficient details of methods and analysis provided to allow replication by others?

Yes

Reviewer Expertise:

biomedical

I confirm that I have read this submission and believe that I have an appropriate level of expertise to confirm that it is of an acceptable scientific standard.

# Reviewer report: Reviewer response for version 3

**DOI:** 10.5256/f1000research.187074.r432149

**Published:** 2025-11-27

**Authors:** Chinmay Bera

**Affiliations:** 1 University of Arkansas for Medical Sciences, Little Rock, Arkansas, USA

This study looked at people in Tunisia living with chronic hepatitis B who were not receiving treatment but had low levels of liver damage when first evaluated. Over several years, about 1 in 6 of these individuals developed worsening liver fibrosis, a sign of liver scarring. The researchers found that people with other health problems—such as diabetes or high cholesterol—and those whose liver enzyme levels increased over time were more likely to develop liver damage. These results support newer international guidelines that recommend offering treatment to more people with hepatitis B to prevent long-term complications like cirrhosis and liver cancer.

 I think the study's strength lies in obtaining liver biopsies to assess fibrosis progression in each participant.

Is the work clearly and accurately presented and does it cite the current literature?

Yes

If applicable, is the statistical analysis and its interpretation appropriate?

Yes

Are all the source data underlying the results available to ensure full reproducibility?

Yes

Is the study design appropriate and is the work technically sound?

Yes

Are the conclusions drawn adequately supported by the results?

Yes

Are sufficient details of methods and analysis provided to allow replication by others?

Yes

Reviewer Expertise:

MASLD and decompensated liver disease. Liver Fibrosis

I confirm that I have read this submission and believe that I have an appropriate level of expertise to confirm that it is of an acceptable scientific standard.

# Reviewer report: Reviewer response for version 3

**DOI:** 10.5256/f1000research.187074.r409443

**Published:** 2025-09-10

**Authors:** Matrujyoti Pattnaik

**Affiliations:** 1 ICMR-Regional Medical Research Centre, Bhubaneswar, Odisha, India

I am satisfied with the authors' response to all reviewer comments, which has significantly improved the clarity and readability of the manuscript.

Is the work clearly and accurately presented and does it cite the current literature?

Partly

If applicable, is the statistical analysis and its interpretation appropriate?

Partly

Are all the source data underlying the results available to ensure full reproducibility?

Yes

Is the study design appropriate and is the work technically sound?

Yes

Are the conclusions drawn adequately supported by the results?

Yes

Are sufficient details of methods and analysis provided to allow replication by others?

Yes

Reviewer Expertise:

Infectious Diseases, Microbiolgy, Public Health

I confirm that I have read this submission and believe that I have an appropriate level of expertise to confirm that it is of an acceptable scientific standard.

# Reviewer report: Reviewer response for version 2

**DOI:** 10.5256/f1000research.182035.r398714

**Published:** 2025-08-13

**Authors:** Matrujyoti Pattnaik

**Affiliations:** 1 ICMR-Regional Medical Research Centre, Bhubaneswar, Odisha, India

**Overall Comment:**

 This retrospective longitudinal cohort study followed 97 untreated chronic HBV patients in Tunisia’s “indeterminate phase” for a median of ~105 months to identify predictors of fibrosis progression. Fibrosis progression occurred in 16.5% of patients, with comorbidities (OR ≈ 53.345) and fluctuations/elevations in ALT (OR ≈ 8.539) as independent risk factors.

**Specific Comments:**

 1. The introduction needs a more logical flow, clearer definitions of terms and a concise articulation of the research gap. Also please explain more about the rationale of the study in the introduction section.

 2. Clarify whether viral load categories are in IU/mL or copies/mL and ensure consistency throughout the manuscript.

 3. “Fluctuating” should be corrected to “Fluctuating.” in Table 2.

 4. Viral load is presented as a mean (8,182,122.4 IU/mL) with excessive decimal accuracy, which is most likely influenced by outliers. Instead, report the median and interquartile range (IQR).

 5. While HBV testing procedures are extensively documented, the cut-off and standardization of viral load tests during a 14-year period may have differed. Please explain whether the assays varied during the research and, if so, how the data were standardized.

 6. Please specify how the missing data and confounding factors were handled during the statistical analysis.

 7. Discuss why comorbidities emerged as a key predictor in your study when few other studies have explored this link.

 8. Discuss your findings with potential confounding from metabolic-associated fatty liver disease (MAFLD).

 9. Please proof check the English in the manuscript to ensure consistency in tenses, minor typo and proper phrasing of sentences.

Is the work clearly and accurately presented and does it cite the current literature?

Partly

If applicable, is the statistical analysis and its interpretation appropriate?

Partly

Are all the source data underlying the results available to ensure full reproducibility?

Yes

Is the study design appropriate and is the work technically sound?

Yes

Are the conclusions drawn adequately supported by the results?

Yes

Are sufficient details of methods and analysis provided to allow replication by others?

Yes

Reviewer Expertise:

Infectious Diseases, Microbiolgy, Public Health

I confirm that I have read this submission and believe that I have an appropriate level of expertise to confirm that it is of an acceptable scientific standard, however I have significant reservations, as outlined above.

# Response

**Published:** 2025-08-22

**Authors:** Sana Rouis

**Affiliations:** Faculty of Medicine of Sousse, Tunisia

Thank you very much for your constructive comments and valuable suggestions regarding our manuscript. We have taken each of your remarks into account and made the necessary revisions. Here is a summary of the changes made:

- We have revised the introduction to improve its logical flow and clarity. Key terms have been defined, and we have articulated the research gap more clearly. Additionally, we have included a detailed explanation of the rationale for the study.
- We clarified that the viral load categories are expressed in IU/mL and ensured consistency in terminology throughout the manuscript.
- The term "Fluctuating" has been corrected in Table 2 as you suggested.
- We modified the presentation of the viral load to report the median and interquartile range (IQR) instead of the mean, to reduce the influence of outliers.
- We added a section explaining that the testing procedures and cut-off values for viral load may have varied over the 14-year period. We also specified how the data were standardized to ensure consistency.
- We provided details on how missing data and confounding factors were handled during our statistical analysis.
- We expanded the discussion on comorbidities, explaining why they emerged as a key predictor in our study, referencing existing studies that have explored this link.
- We included a discussion of our findings considering the potential confounding from metabolic-associated fatty liver disease (MAFLD).
- We conducted a thorough proofreading of the manuscript to ensure consistency in tenses, correct minor typos, and improve sentence phrasing.

We hope that these revisions meet your expectations and enhance the quality of our manuscript. Thank you once again for your time and effort in reviewing our work.

# Reviewer report: Reviewer response for version 2

**DOI:** 10.5256/f1000research.182035.r385856

**Published:** 2025-06-13

**Authors:** Philippa C Matthews

**Affiliations:** 1 The Francis Crick Institute, London, UK

I have re-read the report with revisions. There are important data here which align with new WHO guidelines and the recommendations to relax treatment eligibility criteria. Longitudinal data for HBV are important and this study has merits in providing insights into progressive liver disease in a population from which data are otherwise very limited.

 Before this paper can be published, the authors need to recognise that the landscape has completely changed since they started their study.

 The context needs to be updated - the new WHO treatment guidelines (published in 2024) suggest many more are treated and the 'indeterminate phase' or 'immune tolerant phase' are no longer recommended as a way to describe HBV infection phenotypes. This language should be removed, or at least the terms explained as those in previous use (when the study was developed) rather than suggesting that these are in use and relevant now.  

 The conclusion of the abstract that there is a 'potential rationale for extending therapeutic indications' has  already happened - the authors could say this and discuss how their findings support this change in the landscape (new WHO guidelines, and many others - eg EASL have recently followed suit). 

 While it is reasonable to have conducted an analysis under the umbrella of old guidelines (now superceded), this should be clearly acknowledged - as it stands, the authors appear to have no sight of the biggest shift in treatment guidelines for over a decade. 

 Person-first language should be used, eg rather than 'HBsAg-positive patients' this should be 'people living with Hepatitis B'. 

 Some minor points:

 There are some typos and missing words, such that the whole article needs to be checked for language.

 Table 1 refers to viral load both in copies/ml and in IU/ml - these are two different quantitations, which one is correct?

 The text reports a 'mean' initial viral load of 8,182,122.4 IU/mL - this is extremely skewed by high values and it would be much more helpful to report a median. I do not know of any assay platforms that measure VL down to less than the nearest 1 iu/ml - a decimal point in this number is not appropriate! - stick to the median.

 In the outcomes section of the results, the authors say that 'Antiviral therapy with nucleotide/nucleoside analogues was initiated', but not at what point in the follow up of 105 months this happened - this is an important detail needed to inform understanding of treatment outcomes. 

Is the work clearly and accurately presented and does it cite the current literature?

Partly

If applicable, is the statistical analysis and its interpretation appropriate?

Partly

Are all the source data underlying the results available to ensure full reproducibility?

Partly

Is the study design appropriate and is the work technically sound?

Partly

Are the conclusions drawn adequately supported by the results?

Partly

Are sufficient details of methods and analysis provided to allow replication by others?

No

Reviewer Expertise:

viral hepatitis, infectious diseases epidemiology, hepatitis B treatment, viral hepatitis in Africa, infection biomarkers, risk strratification,

I confirm that I have read this submission and believe that I have an appropriate level of expertise to confirm that it is of an acceptable scientific standard, however I have significant reservations, as outlined above.

# Response

**Published:** 2025-08-22

**Authors:** Sana Rouis

**Affiliations:** Faculty of Medicine of Sousse, Tunisia

We thank the reviewer for their constructive and detailed comments. We have made the following changes to the manuscript in response to your comments:

  

- We have updated the study background to acknowledge the new WHO guidelines published in 2024 and clarified that terms such as "immunotolerant phase" are used in this manuscript to reflect current practices at the time of study design.
- The conclusion of the abstract has been amended to acknowledge that therapeutic indications have already been expanded, and our results support these changes.
- We have used person-centered language throughout the manuscript.
- The units of measurement for viral load in Table 1 have been standardized, and the mean viral load has been replaced with the median with IQR.
- We have added clarification on when antiviral treatment was initiated during follow-up.
- A complete review was conducted to correct typos and inconsistencies.

We hope that these changes meet the reviewer's expectations and improve the quality of the manuscript.

# Response

**Published:** 2025-08-22

**Authors:** Sana Rouis

**Affiliations:** Faculty of Medicine of Sousse, Tunisia

We thank the reviewer for their constructive and detailed comments. We have made the following changes to the manuscript in response to their comments:

- We have updated the study background to acknowledge the new WHO guidelines published in 2024 and clarified that terms such as "immunotolerant phase" are used in this manuscript to reflect current practices at the time of study design.
- The conclusion of the abstract has been amended to acknowledge that therapeutic indications have already been expanded, and our results support these changes.
- We have used person-centered language throughout the manuscript.
- The units of measurement for viral load in Table 1 have been standardized, and the mean viral load has been replaced with the median with IQR.
- We have added clarification on when antiviral treatment was initiated during follow-up.
- A complete review was conducted to correct typos and inconsistencies.

We hope that these revisions meet your expectations and enhance the quality of our manuscript. Thank you once again for your time and effort in reviewing our work.

# Reviewer report: Reviewer response for version 1

**DOI:** 10.5256/f1000research.172481.r362298

**Published:** 2025-04-26

**Authors:** Hela Gdoura

**Affiliations:** 1 Gastro-enterology department, Hedi Chaker University Hospital, Sfax, Tunisia

1/Methods section :

*Strengths:

1. **retrospective cohort study** with **active follow-up of patients** from 2008 to 2022.
2. **Comprehensive clinical follow-up:**
  The follow-up protocol is detailed and rigorous—monitoring viral load, ALT every 6 months, FibroScan, liver biopsies, and clinical events. This indicates **high-quality clinical data**.
3. **Clear definitions of clinical outcomes:**
  Fibrosis progression, cirrhosis, and HCC are **well-defined** using objective criteria. This enhances the **reliability and reproducibility** of the study outcomes.
4. **Inclusion of relevant comorbidities:**
  The analysis includes metabolic factors such as BMI, steatosis, diabetes, etc., which are **clinically relevant** and allow for a more robust multivariate analysis.

* **Points to Clarify or Improve:**

1. **Clarify the study design in the methods/introduction section:**
  The study should be clearly described as a **retrospective longitudinal cohort study**, not cross-sectional

2/Results section:

**Clarity and alignment with the study objective:**

 the main objective was to **identify factors associated with liver fibrosis progression** in patients with **chronic HBV infection in the indeterminate phase**, who were **initially untreated**.

 You have clearly achieved this goal:

1. **Well-defined target population:**
  - You selected patients strictly in the indeterminate phase, with inclusion criteria that matched the definition: HBsAg+, ALT within normal range, viral load > 2000 IU/mL, untreated, and initial fibrosis score F0/F1.
  - Exclusion of patients with low viral load or cirrhosis enhances the internal validity of your findings.
2. **Assessment of the primary outcome:**
  - **Fibrosis progression** was carefully documented (16.5% of patients), including the average time to progression (~71 months).
  - Other relevant outcomes (cirrhosis, HCC, death) were also reported, though they were secondary to your main aim.
3. **Identification of associated factors (statistical rigor):**
  - You conducted both **univariate and multivariate analyses** to identify potential predictors.
  - In the **multivariate logistic regression**, you found **two independent predictors** of fibrosis progression:
    - Presence of **comorbidities** (OR > 50, p < 0.001)
    - **Fluctuations/elevation in ALT** levels during follow-up (OR ~8.5, p < 0.001)
  - The initial viral load, while significant in univariate analysis, **was not an independent factor** — a clinically meaningful conclusion.
4. **Use of quantitative tools:**
  - You used **AUROC** to evaluate the predictive value of the initial viral load and provided cutoff performance data (sensitivity/specificity), which enhances the interpretation of your results — even if this factor was not retained in the final model

* **A few suggestion:**

**On the “indeterminate phase” itself:** Your findings suggest that this phase is **not always benign**, especially in patients with comorbidities or biochemical fluctuations. This is a valuable clinical insight worth highlighting.

 3/discussion part:

*Strengths :

1. **You fully address your research question**
  ✔️ You successfully explore the **factors associated with liver fibrosis progression** in patients in the indeterminate phase of chronic HBV.
  ✔️ The key findings (comorbidities and ALT fluctuations) are clearly interpreted and discussed based on your statistical analysis.
2. **You contextualize your results very well within the literature**
  ✔️ You compare the frequency of the indeterminate phase in your cohort with data from other countries (USA, China, Taiwan), providing a solid background.
  ✔️ Your fibrosis progression rate (16.5%) is consistent with findings from other studies (15–24%), which strengthens the external validity of your results.
  ✔️ You acknowledge discrepancies with previous studies (e.g., those linking low viral load to more fibrosis) and offer thoughtful explanations.
3. **You bring original value with the analysis of comorbidities**
  ✔️ The fact that comorbidities (diabetes, hypertension, dyslipidemia, etc.) are strongly associated with fibrosis progression is a novel and important **contribution**, since these factors are rarely addressed in HBV fibrosis studies.
  ✔️ You correctly highlight that many previous studies excluded patients with metabolic disorders — making your findings all the more relevant.
4. **You clearly explore clinical implications**
  ✔️ You propose a **threshold viral load (8090 IU/mL)** that may guide treatment decisions in indeterminate patients — this is both practical and backed by your data.
  ✔️ You relate your findings to existing treatment recommendations and ongoing debates regarding earlier antiviral therapy.
5. **You acknowledge your study's limitations properly**
  ✔️ You openly address key limitations: loss to follow-up and the absence of HBV genotyping —

5.

* **Suggestions for Minor Improvements:**

1. **On the interpretation of initial viral load**
  ❗️While a viral load > 8090 IU/mL was associated with fibrosis progression in univariate analysis, it was **not significant in multivariate analysis**.
   You might want to clarify that although it’s a potential indicator, it **isn’t an independent predictor**, and thus should not be used in isolation for clinical decisions.
2. **On the link between comorbidities and fibrosis: cautious interpretation**
  ❗️You demonstrate a strong association, but causality isn’t established.
  �� A brief sentence such as *"Further prospective studies are needed to explore the causal relationship..."* would add scientific nuance and reinforce your credibility.
3. **On low rates of HCC and cirrhosis**
  ✔️ The low incidence in your cohort is expected, given the **young average age (32 years)**.
  �� You could emphasize this point more clearly to explain the contrast with higher complication rates in older or Asian cohorts.

Is the work clearly and accurately presented and does it cite the current literature?

Partly

If applicable, is the statistical analysis and its interpretation appropriate?

Yes

Are all the source data underlying the results available to ensure full reproducibility?

Partly

Is the study design appropriate and is the work technically sound?

Yes

Are the conclusions drawn adequately supported by the results?

Yes

Are sufficient details of methods and analysis provided to allow replication by others?

Partly

Reviewer Expertise:

gastroenterology and hepatology

I confirm that I have read this submission and believe that I have an appropriate level of expertise to confirm that it is of an acceptable scientific standard, however I have significant reservations, as outlined above.

# Response

**Published:** 2025-05-13

**Authors:** Sana Rouis

**Affiliations:** Faculty of Medicine of Sousse, Tunisia

Dear Reviewer, thank you for taking the time to provide your valuable feedback and for your thoughtful comments on our work. We greatly appreciate your insights and have carefully considered your suggestions.

**Response to Reviewer 2:**

- **Methods – Study Design:** We thank the reviewer for pointing out the error in the study design description. We have corrected it to "retrospective longitudinal cohort study."
- **Results – Indeterminate Phase & Comorbidities:** We agree with the reviewer's assessment of the clinical significance of our findings regarding the indeterminate phase and the importance of comorbidities. We have emphasized these points in the Discussion section.
- **Discussion – Initial Viral Load & Causality:** We have clarified the interpretation of the initial viral load, acknowledging that it is a potential indicator but not an independent predictor. We have added a sentence to the Discussion emphasizing the need for prospective studies to explore the causal relationship between comorbidities and fibrosis.

I sincerely appreciate the time and care you’ve taken to provide such a comprehensive and insightful review of my work. Your detailed feedback has been immensely helpful, and I’m truly grateful for the effort you’ve made to point out both the strengths and areas that need improvement. I’ve thoughtfully considered each of your suggestions and have done my best to address them in this response. Your valuable insights have not only enhanced this project but will also serve as a guide for my future endeavors. I’m deeply thankful for your expertise and constructive approach. Once again, thank you for your time and support—it means a lot to me.

# Reviewer report: Reviewer response for version 1

**DOI:** 10.5256/f1000research.172481.r362302

**Published:** 2025-01-27

**Authors:** Philippa C Matthews

**Affiliations:** 1 The Francis Crick Institute, London, UK

**Overall comments:**

 This paper sets out to tackle a relevant clinical and public health question, focusing on the extent to which people living with HBV, but not eligible for antiviral therapy, are at risk of progressive liver disease. The authors present an analysis of a subgroup of 97 people living with chronic hepatitis B under follow up in a Tunisian hospital, over a total time period of 14 years, identifying factors associated with progressive liver disease in this untreated population, which include comorbidities and viral load.

**Overall comments:**

1. The authors do not recognise or acknowledge the very major change in the treatment landscape for HBV which has occurred as a result of the release of new high profile HBV data and treatment guidelines released by the World Health Organization in March 2024; these documents should be included as important citations. Thus, the whole paper is out of date and does not apply relevant current treatment thresholds and criteria. While I recognise that the majority of the work and data collection was done preceding these new guidelines, it is nevertheless crucial for the authors at least to describe and recognise the shift in the treatment landscape, and to discuss how the application of new guidelines would modify their conclusions. Likewise, the comorbidity data they have collected are highly pertinent to the new global landscape of metabolic liver disease, MASLD, which should be described and referenced.
2. The methods are lacking a lot of essential details regarding cohort characteristics / location, laboratory methods, thresholds and definitions – this work as it stands is not reproducible.
3. Overall, the paper contains many typographic errors, inconsistent use of language and acronyms, outdated terminology and technical ambiguities; I have captured as many of these as possible in a list of more specific comments which follows.

**Specific comments:**

- The global reports and data should all be updated – rather than citing 2017 global epidemiology estimates, this should refer to the high profile report for viral hepatitis published by the WHO in 2024 and estimates published since 2017.
- Abbreviations should all be reviewed for consistency, and acronyms spelt out in full at first use.  Terminology should be altered to align to internationally recognised nomenclature (for example, change ‘Ag HBs (+)’ to ‘HBsAg+’ and ALAT to ALT (alanine transferase), ‘UI/l’ units should be expressed as IU/L (international units per litre). ‘VHB’ and ‘HBV’ are used interchangeably. 
- The use of the term ‘cytolysis’ is not clear – does this mean the appearance of hepatic necrosis on liver biopsy? Or elevated liver enzymes? Or something else? – this term is not in routine use and I suggest change it to reflect standard terminology in the field.

**Abstract:**

- Abstract should state country/region of work rather than just stating the name of the hospital (most readers, including me, have to look this up to find where it is). It would also be helpful to modify the title of the paper to state the location/region represented by the study, and to provide context by stating the WHO region represented.
-  ‘sensibility of 70.3%’ – should this read sensitivity?
- The abstract states that univariate and multivariate analysis was undertaken but only presents results from univariate analysis.

**Introduction**

-  ‘Severity of liver histological lesions’ is an inaccurate description – the inflammatory and fibrotic changes associated with HBV do not typically represent ‘lesions’ (this language suggests progression to malignancy) – suggest the authors reference appropriate histopathology scoring systems.
- ‘However, the modalities of monitoring its rhythm are not well established’ – this is not true; there are very well established national and international guidelines, algorithms and thresholds.
- ‘ethylism’ – I assume this is referring to alcohol intake? Please clarify.
- ‘Anti-viral treatment with analogues’ – there is a crucial missing word here – therapy is based on ‘nucleotide/nucleoside analagues’

**Methods**

- The reporting of study methods is extremely minimal. The authors should start with a clear description of the nature of the service and population, to include where is the hospital (city, country, WHO region)? what it the population catchment, who is referred, is this adults only or also adolescents/children, who refers patients, and on what basis are they referred to this centre? What is the overall context of healthcare provision, including state funding/out of pocket? Where were lab assays undertaken, on what laboratory platforms (need details of manufacturers, reagents, thresholds, reporting, validation).
- What were the criteria for liver biopsy?
- What treatment criteria or guidelines were in use during the period of the study? What antiviral treatment is used in this setting?
- The authors state that their study meets STROBE criteria, but this checklist has not been provided – suggest add as supplementary data.

**Terminology**

- The term ‘immunotolerant’ is recognised to be misleading and is not featured in updated guidelines. However, if it is applied to this historic cohort, then it needs a clear definition, supported by citations.
- Definition of chronic alcoholism as ‘two to three standard glasses per day’ is very vague. ‘Standard glasses’ of what? How much is ‘a glass’? – alcohol intake is conventionally measured in units or grams. Having defined alcohol intake, it should then be reported in the cohort characteristics or results.
- What reference ranges have been applied to lab parameters? How often were measurements repeated? How are ‘normal’ / ‘high’ / ‘fluctuating’ defined for ALT and viral load, respectively?
- ‘Qualitative variables are expressed as percentages’ seems likely to be incorrect – calculation of a percentage suggests data are quantitative.
- How were dyslipidaemia and hypertension diagnosed/defined?

**Results**

- The metadata table on Figshare needs some annotation to ensure variables are presented clearly, with units, that coding is clear and that blank cells are explained (is this missing data? Or does a blank cell mean ‘no’ or ‘zero’?)
- Changes in ‘ALAT rates’ does not make sense – should this be changes in absolute value of ALT?
- ‘An initial viral load of 8090 UI/l was associated with the progression of fibrosis with a sensitivity of 70.3% and specificity of 63%’ – it is not clear how this specific viral load figure was chosen or optimised as a threshold. Should this be presented as a baseline viral load *above* this threshold?
- FIB-4 and APRI are both mentioned as potential modalities to assess fibrosis but these data have not been presented – please explain why not. AST and platelet count both appear in the metadata table.

**Tables and Figures**

- Viral loads in table 1 are defined as ‘moderate’ and ‘high’ but these need to be defined by a quantitative threshold, ideally aligning with standard thresholds used in the field.
- Viral loads appear to be given to one decimal place, but I am not aware of any assays that report to a resolution below the nearest 1 IU/L – please clarify.
- Table 1: check for typos and inconsistent units, abbreviations and terminology. In this table it is not clear whether the number reported for liver biopsy and fibroscan are the number who had these tests or the number for whom the tests were abnormal? If the former, the actual results of the investigation should be reported as well.
- **Table 2** – all thresholds need to be defined

- Figure 2 is annotated in French; to be concordant with the rest of the article, this should  be translated to English.

**Limitations**

- The authors should include the problem of a modest number of patients in their population of interest. These small numbers mean that confidence intervals around estimates are extremely wide, and that there are very small numbers of patients (only one or two) who experienced clinical outcomes of interest, even over a long time period of observation.
- The authors should discuss why they collected data for specific comorbidities and not others (eg. why not other cardiovascular parameters? And/or renal disease?), and discuss whether it is reasonable to take the approach they have used in analysing these together as a single group.  
- I am not convinced that this analysis of a small number of individuals with very few clinically relevant endpoints adds anything to the field that is not already known. However, recognising that data for the whole African continent are lacking, and that we need better understanding of outcomes of HBV infection in diverse cohorts, the paper could potentially be approved and indexed with major revisions to tackle the comments above.

Is the work clearly and accurately presented and does it cite the current literature?

Partly

If applicable, is the statistical analysis and its interpretation appropriate?

Partly

Are all the source data underlying the results available to ensure full reproducibility?

Partly

Is the study design appropriate and is the work technically sound?

Partly

Are the conclusions drawn adequately supported by the results?

Partly

Are sufficient details of methods and analysis provided to allow replication by others?

No

Reviewer Expertise:

viral hepatitis, infectious diseases epidemiology, hepatitis B treatment, viral hepatitis in Africa, infection biomarkers, risk strratification,

I confirm that I have read this submission and believe that I have an appropriate level of expertise to state that I do not consider it to be of an acceptable scientific standard, for reasons outlined above.

# Response

**Published:** 2025-05-13

**Authors:** Sana Rouis

**Affiliations:** Faculty of Medicine of Sousse, Tunisia

We sincerely thank the reviewer for his thorough and insightful comments, which have significantly strengthened our understanding of the manuscript's strengths and areas for improvement. We appreciate the reviewer's recognition of the study's relevance and the valuable clinical insights it provides. We have carefully addressed each comment and incorporated the suggested revisions into the manuscript. A revised manuscript is included with this response.

**Response point by point: **

- **General Comments – Treatment Landscape & Guidelines:** We acknowledge the reviewer's valid point regarding the evolving treatment landscape for HBV and the release of the 2024 WHO guidelines. We have added a paragraph to the Introduction and Discussion sections explicitly addressing this shift, discussing how the application of new guidelines would modify our conclusions and highlighting the importance of our findings in contexts where treatment access may be limited. We have also included citations to the 2024 WHO report and other relevant publications. We have also added a discussion of MASLD and its relevance to our patient population. We have also modified the manuscript title according to the reviewer’s proposition.

- **General Comments – Reproducibility & Terminology:** We agree with the reviewer's concerns regarding the lack of detail in the Methods section and the inconsistent use of terminology. We have significantly expanded the Methods section to provide a more comprehensive description of the study setting, patient population, laboratory methods (including manufacturer details, reagents, and reporting thresholds), and statistical analysis. We have also standardized terminology throughout the manuscript, replacing "cytolysis" with more precise language and correcting abbreviations (e.g., HBsAg+, ALT).
- **Abstract – Country/Region & Sensitivity:** We have updated the Abstract (and also the methodology section) to specify the country (Tunisia) and region (WHO Eastern Mediterranean Region) of the study. We have also corrected "sensibility" to "sensitivity."
- **Introduction – Liver Lesions & Monitoring:** We have revised the language regarding "severity of liver histological lesions" to avoid the implication of malignancy and have replaced it with more appropriate terminology. We have also corrected the statement regarding monitoring modalities, acknowledging the well-established national and international guidelines. We have clarified the meaning of "ethylism" as alcohol intake.
- **Methods – Study Design, Laboratory Details, Treatment Guidelines, STROBE Checklist:** We have clarified the study design as a retrospective longitudinal cohort study. We have significantly expanded the description of the laboratory methods, including details on the platforms and reagents used. We have specified the treatment guidelines in use during the study period (EASL guidelines). We have included the STROBE checklist as supplementary data.
- **Terminology – Immunotolerant, Alcohol Intake, ALT/Viral Load Definitions:** We have addressed the term "immunotolerant" by providing a clear definition and relevant citations. We have clarified the definition of alcohol intake, specifying units and grams. We have defined the thresholds used for classifying ALT and viral load as normal, high, or fluctuating.
- **Results – ALT Rates & Viral Load Cutoff:** We have corrected "ALAT rates" to "absolute ALT value." We have clarified the rationale for the viral load cutoff of 8090 IU/mL and presented it as a baseline viral load *above* this threshold.
- **Tables & Figures – Thresholds, Units, Figure Annotations:** We have ensured that all thresholds are clearly defined in the tables. We have verified the units and corrected any inconsistencies. We have translated the figure 2 annotations to English.
- **Limitations – Patient Numbers:** We have added a discussion of the limitations posed by the modest number of patients in the study and the resulting wide confidence intervals. We have also expanded on the discussion of causality regarding comorbidities.

Finally, thank you for taking the time to provide such a thorough and thoughtful review of my work. Your detailed feedback has been incredibly valuable, and I truly appreciate the effort you’ve put into highlighting both the strengths and areas for improvement. I’ve carefully considered each of your points and have addressed them to the best of my ability in this response. Your insights have not only helped refine this work but will also guide my future efforts. I’m grateful for your expertise and constructive approach, which have significantly contributed to the quality of this project. Thank you once again for your time and support.
